# Genetically Induced Mouse Model for Colon-specific Epithelial Cell Tumorigenesis Driven by Loss of K8 and Apc

**DOI:** 10.1016/j.jcmgh.2025.101716

**Published:** 2025-12-24

**Authors:** Mina Tayyab, Mira M.E. Minkkinen, Carl-Gustaf A. Stenvall, Lauri Polari, Victor Nielsen, Yatrik M. Shah, Diana M. Toivola

**Affiliations:** 1 Department of Natural and Health Sciences, Cell Biology, Faculty of Science and Engineering, Åbo Akademi University, Turku, Finland; 2 InFLAMES Research Flagship Center, Åbo Akademi University, Turku, Finland; 3 Department of Pathology, Wellbeing Service County of Satakunta, Pori, Finland; 4 Department of Molecular and Integrative Physiology, University of Michigan, Ann Arbor, Michigan; 5 Department of Internal Medicine, University of Michigan, Ann Arbor, Michigan; 6 Turku Center for Disease Modeling, Turku, Finland

**Keywords:** CDX2, Colorectal Cancer, Keratin 8, Mouse Model

## Abstract

**Background & Aims:**

Loss of keratin 8 (K8) has been shown to increase susceptibility towards colonocyte hyperproliferation and tumorigenesis. However, most colorectal cancer (CRC) mouse models require carcinogen, develop small intestinal tumors, or have a long latency period. The aim was to establish a genetic, colon-specific, and more human-like CRC model driven by loss of K8 and adenomatous polyposis coli (Apc).

**Methods:**

Colon-specific targeting using CDX2P-CreER^T2^ mice was used to generate K8^flox/flox^; CDX2P-CreER^T2^ and K8^flox/flox^; CDX2P-CreER^T2^; Apc^flox/+^ mice. Disease activity was monitored, and colon was analyzed for tumor burden and histopathology over time. Keratin expression, inflammation, proliferation, cell polarity, colonocyte populations, and cell division symmetry were assessed using immunoblotting and immunofluorescence analysis. This data was compared with K8 expression analysis in patients with CRC and in UALCAN database.

**Results:**

K8^flox/flox^; CDX2P-CreER^T2^ mice develop mild diarrhea and express reduced K8 and partner keratins in a mosaic pattern in the colonic epithelium. K8-negative colon areas display increased crypt loss and more inflammation predominantly in the proximal colon. Increased colonocyte proliferation is observed throughout the colon. Impaired cell polarity and higher number of stem and progenitor cells with a shift towards asymmetric cell division in K8-negative areas of the distal colon highlight a pro-tumorigenic environment. Mice with additional monoallelic Apc inactivation show colon tumorigenesis and epithelial to mesenchymal transition distally. In patients with CRC, tumor K8 expression is decreased independent of disease type and stage, age, or gender.

**Conclusions:**

Genetic colon-specific mouse model with loss of K8 and Apc adequately resembles human CRC. This study identifies anti-tumorigenic protective roles of colonocyte K8 in the colon.

## Graphical abstract

**Figure:**
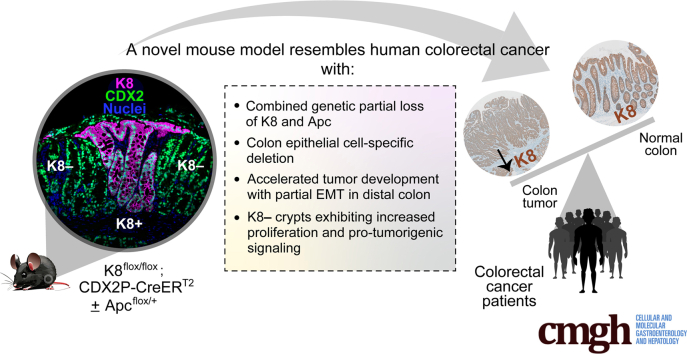

**Summary**

New colon tumorigenesis mouse model is driven by loss of colonocyte keratin 8 (K8) and adenomatous polyposis coli. Pro-tumorigenic changes occur in K8-negative areas, and tumors develop in the colon like in patients with colorectal cancer. Decreased K8 expression is observed in patients with colorectal cancer.

**What You Need to Know**

**Background**

There is a need to develop a human-relevant colorectal cancer (CRC) model without carcinogen induction, extracolonic tumor load, and prolonged latency periods.

**Impact**

The study identifies keratin 8 as dysregulated in CRC and a novel factor in tumor suppression. The model can benefit the research community and the pharmaceutical industry for preclinical studies.

**Future Directions**

This human-relevant model acts as a bridge between preclinical and clinical CRC research and may be used to identify new molecular disease mechanisms and CRC drug targets.

The intestinal epithelium serves as a selectively permeable barrier and exhibits the highest proliferation rate of any tissue, with complete self-renewal occurring approximately every 3 to 5 days in mammals.^1^^,^^2^ Dysregulation of this tightly controlled barrier and rapid cell turnover often predisposes individuals to life-threatening conditions, including inflammatory bowel disease (IBD)^3^ and colorectal cancer (CRC).^4^ CRC is a multifactorial malignancy, and its incidence is rising rapidly, particularly among young adults.^5^ Early detection of CRC and identification of the factors driving its pathogenesis are crucial for guiding treatment strategies. In this regard, experimental models have significantly advanced our understanding of CRC and served as a bridge between preclinical and clinical research. Mouse models are extensively used to study CRC pathogenesis and explore treatment options.^6^ However, these models have several limitations. Many require carcinogenic chemicals, such as azoxymethane (AOM), and/or take a long time to develop tumors.^7^ Additionally, the small intestinal tumor location in adenomatous polyposis coli (Apc) inactivation-based models (such as the Apc Min/+ mutation leading to a truncated protein similar a human germline mutation) is notably different from that in humans, where the primary tumor site is typically the colorectum.^8^ Therefore, there is a need to develop models that more accurately recapitulate human CRC.

In efforts to refine Apc-based mouse models, use of the colon epithelium specific caudal-type homeobox transcription factor 2 promoter (CDX2P) regulatory elements with the loxP-CreER^T2^ system has enabled Apc inactivation in the adult mouse colon epithelium only.^9^^,^^10^ In previous work, we showed that loss of intestinal keratin 8 (K8) makes the colon more susceptible to tumor development upon a second carcinogenic hit with AOM.^11^ In the present study, we aimed to avoid AOM toxicity and develop a genetic mouse model for colon tumorigenesis by inactivating K8 and Apc specifically in colon epithelial cells.

K8 is an integral cytoskeletal component of intestinal epithelial cells. Healthy intestinal epithelium primarily expresses type I (K18–K20) keratins paired with type II (K7–K8) keratins. However, K7 is only expressed in the mouse intestine and is undetectable in the healthy human intestine.^12^^,^^13^ Loss of the major type II keratin K8 in mice dramatically reduces the expression of its type I partners, K18 and K19, and to a lesser extent, type II K7 in the colon. This leads to a colon phenotype characterized by diarrhea, hyperproliferation, epithelial damage, modest inflammation, imbalanced differentiation, and experimentally induced tumorigenesis.^11^^,^^14^^,^^15^ One of the earliest pathological events following K8 loss is colonocyte hyperproliferation.^16^ After K8 loss, proliferative signaling activity is increased in the colonic epithelium.^11^^,^^14^

Given the involvement of K8 in multiple protective roles in the colon, we investigated whether combining the loss of K8 and Apc in colon epithelial cells could enhance and accelerate colon tumorigenesis in mice. To drive the loss of K8 and Apc in colon epithelial cells, we used the CDX2P sequence-dependent loxP-CreER^T2^ system. Our new K8^flox/flox^; CDX2P-CreER^T2^ mouse model displays a patchy, localized loss of K8, accompanied by epithelial changes in the K8-negative areas of the colon. When combined with monoallelic Apc inactivation, these mice exhibit significantly enhanced and accelerated colon tumorigenesis compared with mice with monoallelic Apc inactivation and intact K8. Additionally, human patients with colon adenocarcinoma show decreased expression of K8 in colon tumors. The model we generated is rapid, genetic, and highly relevant to human CRC, as it involves Apc inactivation, reduced K8 expression, and tumor development specifically in the colon. These findings highlight the essential role of colonocyte K8 in maintaining epithelial integrity and protecting against tumorigenesis.

## Results

### Colon-specific K8 Downregulation in Tamoxifen-treated K8^flox/flox^; CDX2P-CreER^T2^ Mice Leads to Mild Diarrhea and Reduced Expression of Major Colonic Keratins

**Figure 1:**
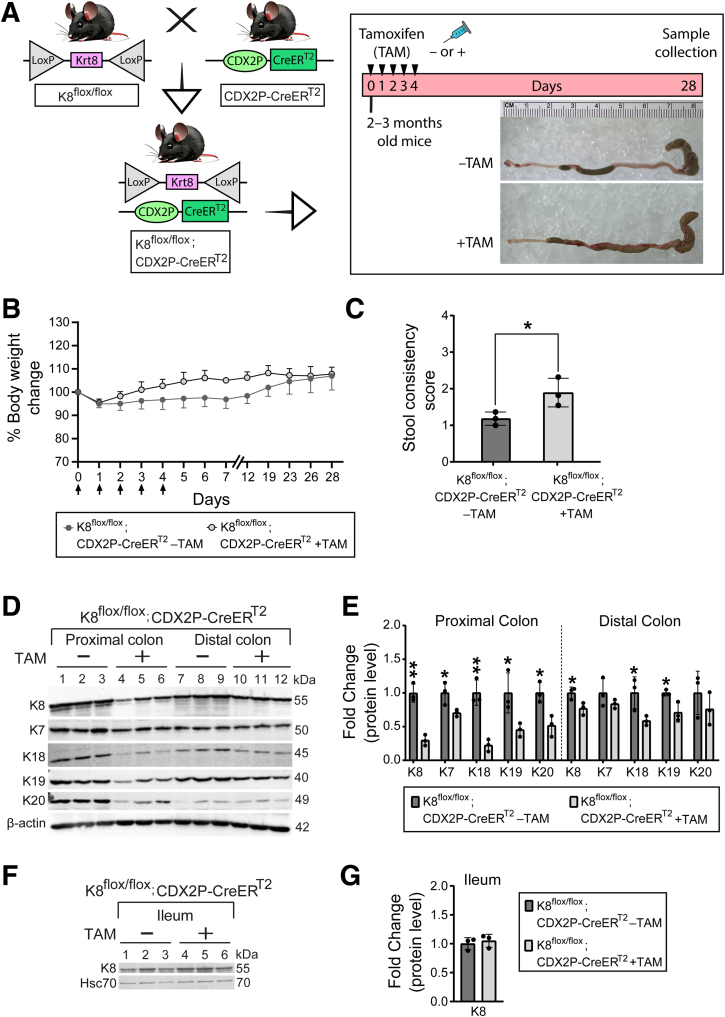
**Colon epithelial cell K8 downregulation in TAM-treated K8^flox/flox^; CDX2P-CreER^T2^ mice leads to mild diarrhea and reduced protein levels of major keratins in the colon.** (*A*) Schematic representation of K8^flox/flox^; CDX2P-CreER^T2^ mouse model generation and experimental study timeline. Adult 2- to 3-month-old mice were administered vehicle (–TAM) or tamoxifen (+TAM), indicated by *black arrowheads*. Representative colon images on day 28. (*B*) Percentage body weight changes of K8^flox/flox^; CDX2P-CreER^T2^ (–TAM/+TAM) mice was determined and presented as mean (n = 3 mice per group) ± SD at different time points during the experimental study; *black arrows* indicate –TAM/+TAM administrations. (*C*) Stool consistency of K8^flox/flox^; CDX2P-CreER^T2^ (–TAM/+TAM) mice was scored and presented as mean (n = 3 mice per group, each data point represents an individual mouse) ± SD. (*D*) Total proximal colon lysates from K8^flox/flox^; CDX2P-CreER^T2^ –TAM (Lanes 1–3), K8^flox/flox^; CDX2P-CreER^T2^ +TAM (Lanes 4–6), and total distal colon lysates from K8^flox/flox^; CDX2P-CreER^T2^ –TAM (Lanes 7–9), K8^flox/flox^; CDX2P-CreER^T2^ +TAM (Lanes 10–12) were immunoblotted for K8, K7, K18, K19, and K20. β-actin was used as the loading control. (*E*) Western blots from (*D*) were quantified and normalized to β-actin. The results are presented as mean (n = 3 mice per group, each data point represents an individual mouse) protein fold changes ± SD. (*F*) Total ileum lysates from K8^flox/flox^; CDX2P-CreER^T2^ –TAM (Lanes 1–3) and +TAM (Lanes 4–6) were immunoblotted for K8 and Hsc70 was used as the loading control. (*G*) Western blot from (*F*) was quantified and normalized to Hsc70 and presented as mean (n = 3 mice per group, each data point represents an individual mouse) protein fold changes ± SD. The statistical significance was determined after performing 2-way ANOVA Bonferroni’s post hoc test for B and unpaired Student’s *t*-test for (*C, E, and G*), shown as ∗*P* < .05 and ∗∗*P* < .01.

The colon epithelium specific targeting of K8 was achieved by crossing mice with floxed Krt8 gene^11^ to mice expressing a tamoxifen inducible CDX2P-CreER^T2^ transgene,^9^ and thereby generating the colon-specific K8^flox/flox^; CDX2P-CreER^T2^ mouse model. Experimental mice received tamoxifen (TAM) to disrupt K8 expression in colon epithelial cells and control mice received the vehicle (corn oil). Mice were monitored for their body weight and stool consistency, and samples were collected on day 28 (Figure 1*A*). There was no difference in body weight of mice between groups by day 28 (Figure 1*B*). However, TAM-treated mice had, on average, looser stool than the vehicle-treated mice (Figure 1*C*). Mean colon length did not differ between TAM-treated mice and vehicle-treated mice (8.3 ± 0.5 vs 7.3 ± 0.2 cm, respectively; *P* = .055).

Proximal and distal colon tissue were analyzed for protein levels of K8, the other type II keratin (K7) and type I keratins (K18, K19, and K20). K8 protein levels were downregulated in both proximal and distal colon, followed by reduced K18 and K19 protein expression levels. However, K7 and K20 protein levels were significantly reduced only in the proximal colon (Figure 1*D* and *E*). In the ileum, K8 protein levels remained unchanged when compared between vehicle-treated and TAM-treated mice as expected (Figure 1*F* and *G*). These findings show that TAM-treated K8^flox/flox^; CDX2P-CreER^T2^ mice display a significant downregulation of K8 in the colon rather than a complete K8 inactivation 28 days after the first TAM administration, with unaltered K8 protein levels in the ileum.

### CDX2P-CreER^T2^-mediated Recombination in Tamoxifen-treated K8^flox/flox^; CDX2P-CreER^T2^ Mice Results in Early Mosaic K8 Disruption with Crypt Loss in K8-negative Areas

**Figure 2:**
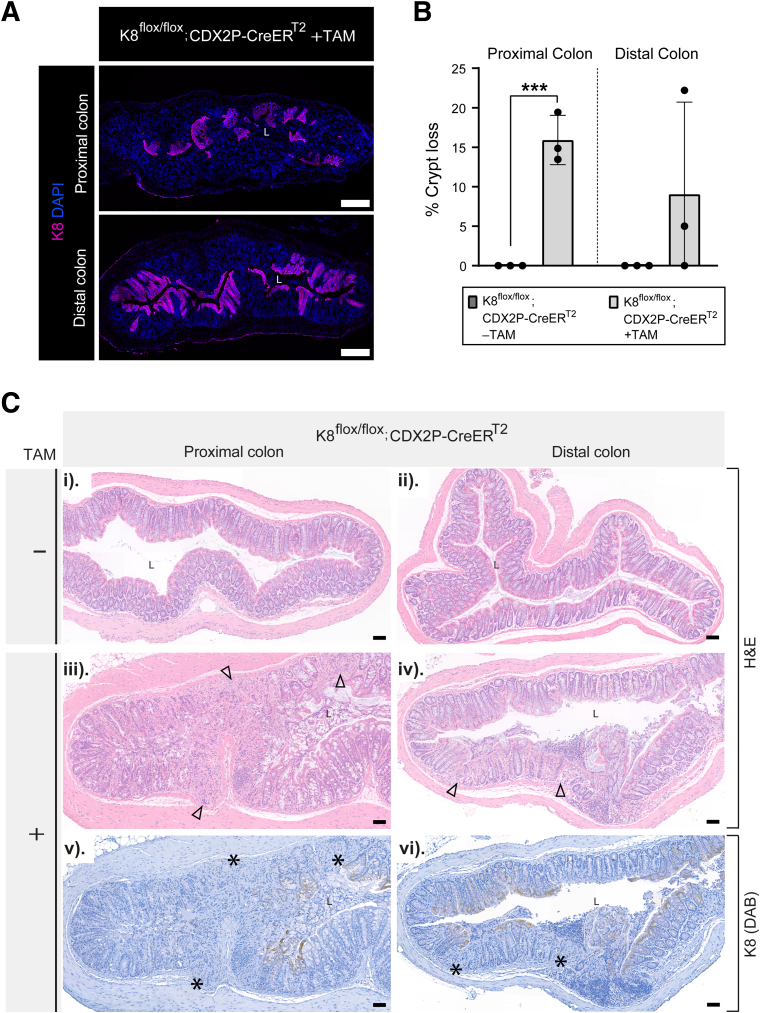
**K8 downregulation in K8^flox/flox^; CDX2P-CreER^T2^ mice shows patchy knockout pattern in the colon, and crypt damage occurs in K8-negative areas.** (*A*) Representation of K8 (*magenta*) expression in entire proximal and distal colon section of K8^flox/flox^; CDX2P-CreER^T2^ +TAM mice (n = 3 mice) on day 28, nuclei, DAPI (*blue*); scale bar = 200 μm. (*B*) Percentage of crypt loss was determined from H&E-stained proximal and distal colon sections of K8^flox/flox^; CDX2P-CreER^T2^ (–TAM/+TAM) on day 28 and presented as mean (n = 3 mice per group, 3 sections per proximal/distal colon) ± SD, each data point represents an individual mouse. (*C*) (*i–iv*) H&E sections from K8^flox/flox^; CDX2P-CreER^T2^ (–TAM/+TAM) mice (n = 3 mice per group) with *black arrowheads* indicating crypt loss in the proximal and distal colon; scale bar = 100 μm. (*v–vi*) K8 (DAB) immunolabeling in the same proximal and distal colon sections of K8^flox/flox^; CDX2P-CreER^T2^ +TAM mice (n = 3 mice) with *black asterisks* indicating K8-negative areas; scale bar = 100 μm. All the images are representative of n = 3 mice per group. The statistical significance was determined after performing unpaired Student’s *t*-test for (*B*), shown as ∗*P* < .05; ∗∗*P* < .01; and ∗∗∗*P* < .001.

**Figure 3:**
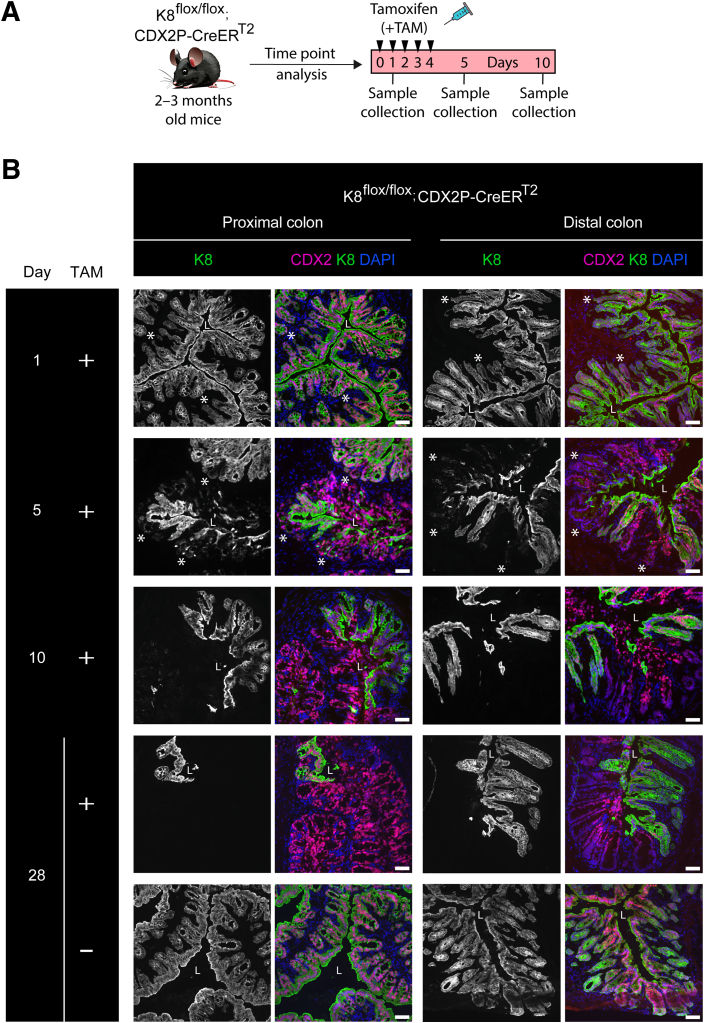
**K8 patchy knockout pattern appears at day 5 in the colon of TAM-treated K8^flox/flox^; CDX2P-CreER^T2^ mice.** (*A*) Schematic representation of time course experiment for K8^flox/flox^; CDX2P-CreER^T2^ mouse model. Adult 2- to 3-month-old mice were administered tamoxifen (+TAM), indicated by *black arrowheads*. Samples were collected on days 1, 5, and 10. (*B*) Immunofluorescence staining of K8 (*green*), CDX2 (*magenta*), and nuclei, DAPI (*blue*) in proximal and distal colon sections of K8^flox/flox^; CDX2P-CreER^T2^ (+TAM for day 1, 5, 10, and –TAM/+TAM for day 28) mice (n = 3 mice per day, and for day 28, n = 3 mice per group) is shown. *Asterisk* points to the reduced K8 expression at crypt bottom on day 1 and patchy loss pattern on day 5; scale bar = 50 μm. All the images are representative of n = 3 mice per day and group.

**Figure 4:**
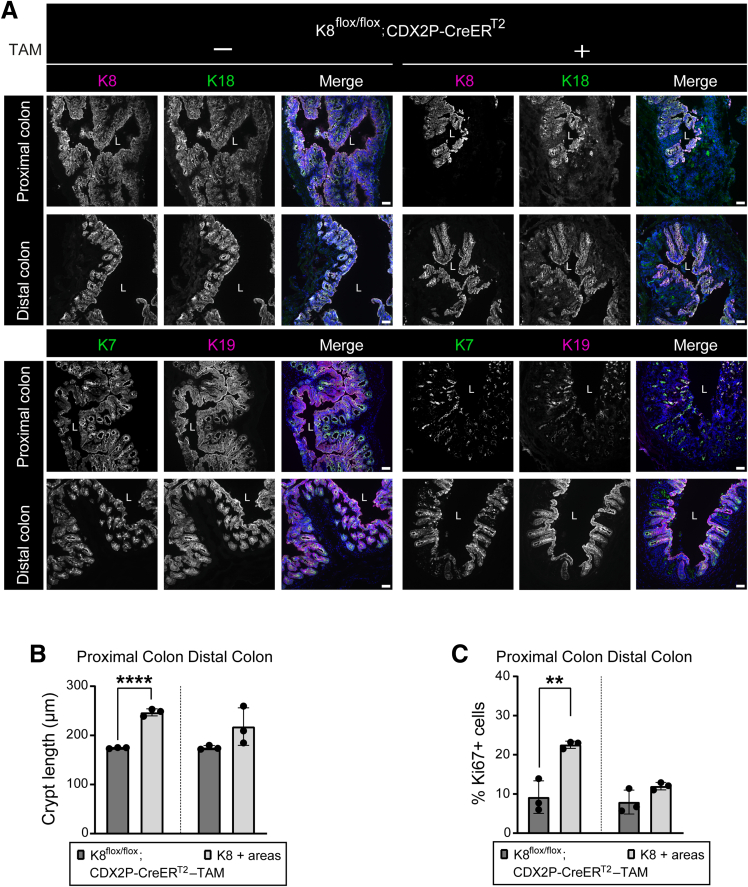
**K8 downregulation in TAM-treated K8^flox/flox^; CDX2P-CreER^T2^ mice also induces patchy expression pattern of major other keratins in the colon.** (*A*) Immunofluorescence staining of K8 (*magenta*) and K18 (*green*) or K19 (*magenta*) and K7 (*green*) with nuclei, DAPI (*blue*) in proximal and distal colon sections from K8^flox/flox^; CDX2P-CreER^T2^ (–TAM/+TAM) mice (n = 3 mice per group) on day 28 is shown; scale bar = 50 μm. All the images are representative of n = 3 mice per group. (*B*) Lengths of K8^flox/flox^; CDX2P-CreER^T2^ –TAM crypts and K8+ crypts of K8^flox/flox^; CDX2P-CreER^T2^ +TAM mice from proximal and distal colon on day 28 were quantified and presented as mean (n = 3 mice per group, 30 –TAM and 15 K8+ crypts per proximal/distal colon) ± SD, each data point represents an individual mouse. (*C*) Percentage of Ki67+ cells in K8^flox/flox^; CDX2P-CreER^T2^ –TAM and K8+ crypts of K8^flox/flox^; CDX2P-CreER^T2^ +TAM mice from proximal and distal colon on day 28 was quantified and presented as mean (n = 3 mice per group, 3 images –TAM and 6–15 K8+ crypts per proximal/distal colon) ± SD, each data point represents an individual mouse. The statistical significance was determined after performing unpaired Student’s *t*-test for (*B* and *C*), shown as ∗*P* < .05; ∗∗*P* < .01; ∗∗∗*P* < .001; and ∗∗∗∗*P* < .0001.

To investigate the observed downregulation and incomplete deletion of K8 in the colon on day 28, we analyzed K8 expression patterns in the colonic epithelium using immunofluorescence staining. K8 inactivation was partial, resulting in a patchy knockout pattern characterized by regions lacking K8 expression (K8-negative) interspersed with regions retaining K8 expression (K8-positive). Notably, the proximal colon exhibited a higher prevalence of K8-negative areas compared with the distal colon (Figure 2*A*). To assess the timeline of the initial downregulation of K8 in the crypts, TAM-treated K8^flox/flox^; CDX2P-CreER^T2^ mice were sacrificed at day 1, 5, and 10 after the first TAM administration (Figure 3*A*). On day 1, K8 expression appeared moderately decreased at the bottom of the crypts in the proximal and distal colon. On day 5 and 10, the patchy pattern of K8 was observed in the proximal and distal colon. However, these crypts still had some remnant K8, which was slightly more on day 5 in comparison to day 10 (Figure 3*B*). Additionally, K7, K18, and K19 demonstrated patchy expression patterns that mirrored K8 staining in both the proximal and distal colon on day 28 (Figure 4*A*).

**Figure 5:**
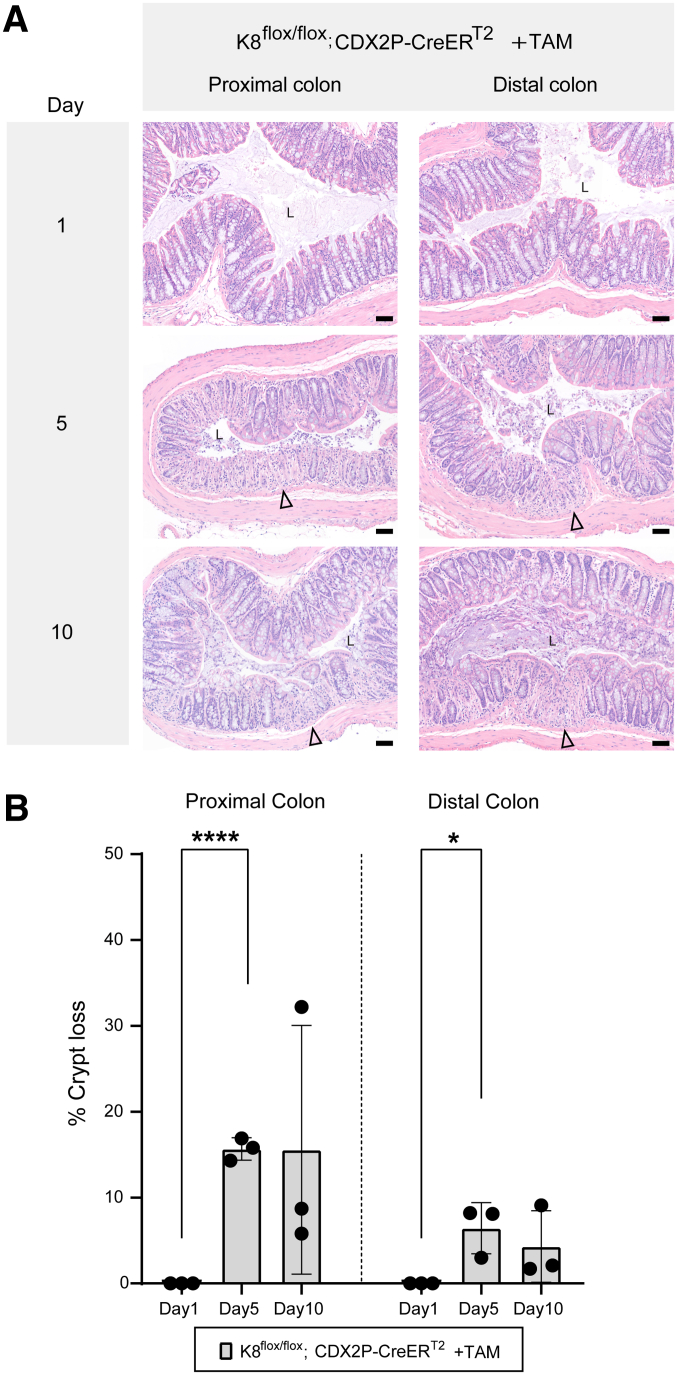
**Crypt damage simultaneously occurs with patchy K8 loss at day 5 in TAM-treated K8^flox/flox^; CDX2P-CreER^T2^ mice.** (*A*) H&E sections from K8^flox/flox^; CDX2P-CreER^T2^ +TAM mice (n = 3 mice per day) with *black arrowheads* indicating crypt loss in the proximal and distal colon; scale bar = 100 μm. All the images are representative of n = 3 mice per day. (*B*) Percentage of crypt loss was determined from H&E-stained proximal and distal colon sections of K8^flox/flox^; CDX2P-CreER^T2^ +TAM and presented as mean (n = 3 mice per day, 1 section per proximal/distal colon) ± SD, each data point represents an individual mouse. All the images are representative of n = 3 mice per day. The statistical significance was determined after performing unpaired Student’s t test for (*B*), shown as ∗*P* < .05; ∗∗*P* < .01; ∗∗∗*P* < .001; and ∗∗∗∗*P* < .0001.

Next the histological phenotype in the colon of TAM-treated K8^flox/flox^; CDX2P-CreER^T2^ mice was assessed, focusing on crypt damage. Mice with K8 downregulation exhibited significantly higher crypt loss in the proximal colon compared with the distal colon, where substantial inter-mouse variability was observed on day 28 (Figure 2*B*). Significant crypt loss occurred on day 5, when crypts also began to show partial loss of K8 (Figure 5*A* and *B*). Importantly, crypt loss was confined to K8-negative regions of the colon (Figure 2*Ciii–vi*). Collectively, these findings indicate that patchy K8 knockout pattern and crypt loss begin to emerge on day 5 following the first TAM administration. K8 downregulation is more pronounced in the proximal colon, which correlates with increased crypt loss, whereas the distal colon exhibits fewer K8-negative areas and less crypt damage.

### K8-negative Areas in the Proximal Colon of Tamoxifen-treated K8^flox/flox^; CDX2P-CreER^T2^ Mice Exhibit Increased Number of Myeloperoxidase-positive Cells

**Figure 6:**
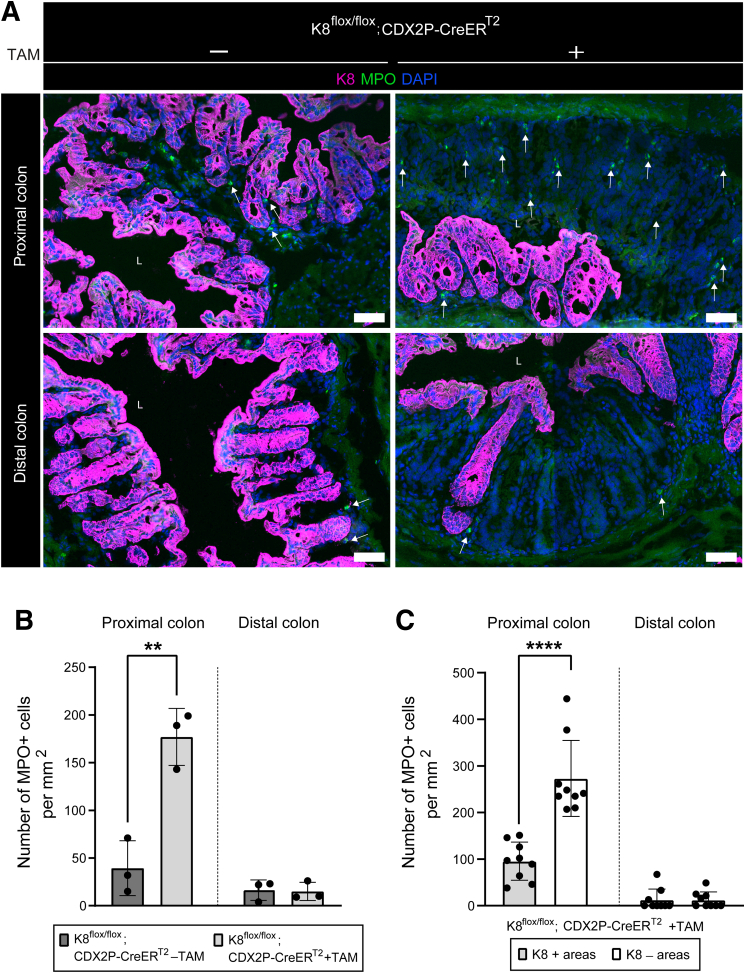
**TAM-treated K8^flox/flox^; CDX2P-CreER^T2^ mice have more MPO+ cells in K8-negative areas of the proximal colon.** (*A*) Immunofluorescence staining of K8 (*magenta*), MPO (*green*), nuclei, DAPI (*blue*) in proximal and distal colon section of K8^flox/flox^; CDX2P-CreER^T2^ (–TAM/+TAM) mice (n = 3 mice per group) on day 28 is shown; *white arrows* indicate MPO+ cells; scale bar = 50 μm. All the images are representative of n = 3 mice per group. (*B*) MPO+ cells within proximal and distal colon mucosal area were quantified from K8^flox/flox^; CDX2P-CreER^T2^ (–TAM/+TAM) mice and presented as mean (n = 3 mice per group, 1 section per proximal/distal colon) ± SD, each data point represents an individual mouse. (*C*) MPO+ cells within K8+ and K8– areas of proximal and distal colon were counted in K8^flox/flox^; CDX2P-CreER^T2^ +TAM mice and results are presented as mean (n = 3 mice, 3 K8+ and 3 K8– areas per proximal/distal colon) ± SD, each data point represents an individual area. The statistical significance was determined after performing unpaired Student’s *t*-test for (*B and C*), shown as ∗*P* < .05; ∗∗*P* < .01; ∗∗∗*P* < .001; and ∗∗∗∗*P* < .0001.

Since crypt loss was localized to K8-negative areas in these mice, we investigated whether colitis develops and whether K8-negative regions promote neutrophil recruitment 28 days after TAM induction of K8 loss. Myeloperoxidase (MPO)-positive cells were quantified in the lamina propria, spanning from the lumen to the muscularis mucosa, in both the proximal and distal colon. Tamoxifen-treated mice exhibited a significant increase in MPO+ cells in the proximal colon compared with vehicle-treated controls, whereas the distal colon showed minimal MPO+ cell infiltration, with no significant difference between treatment groups (Figure 6*A* and *B*). Within the proximal colon of TAM-treated mice, K8-negative areas exhibited significantly higher MPO+ cell infiltration compared with K8-positive areas (Figure 6*C*). In contrast, no differences in MPO+ cell numbers were observed between K8-negative and K8-positive areas in the distal colon (Figure 6*C*). These findings indicate that neutrophil recruitment is selectively increased in K8-negative regions of the proximal colon, suggesting that K8 deficiency drives localized colonic inflammation in this model.

### K8 Loss Predisposes Tamoxifen-treated K8^flox/flox^; CDX2P-CreER^T2^ Mice Towards Colon Hyperproliferation With More Ki67+ cells and Diminished Notch-1 Expression in K8-negative Areas

**Figure 7:**
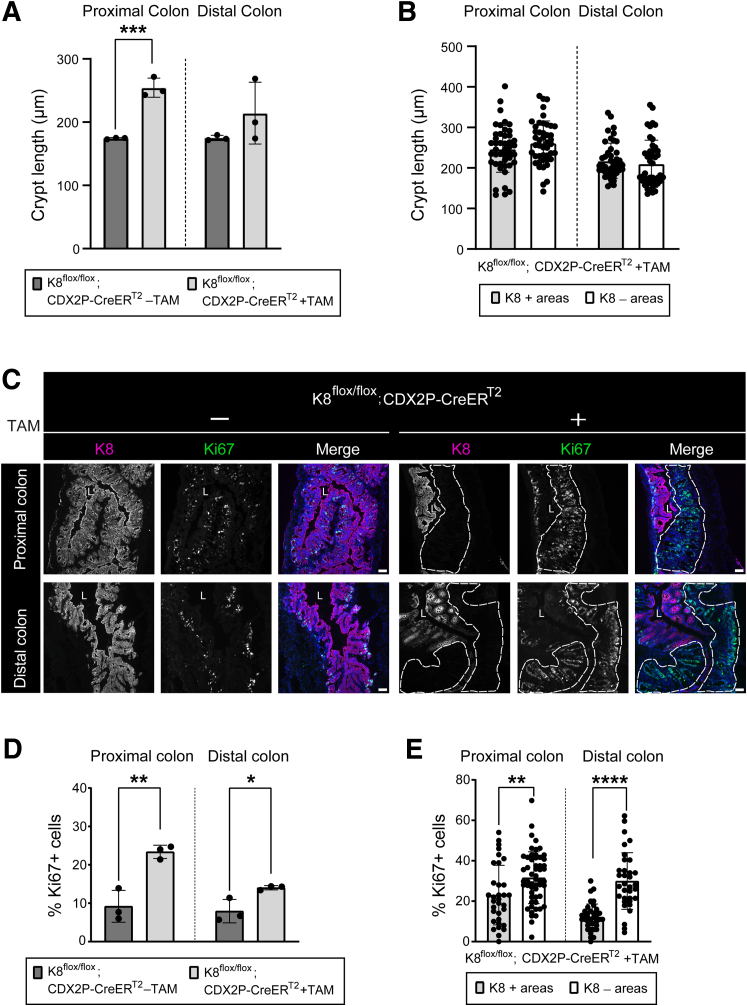
**Colonic K8 downregulation based K8^flox/flox^; CDX2P-CreER^T2^ mice have lengthened crypts and more Ki67+ cells in the colon.** (*A*) Crypt lengths from proximal and distal colon of K8^flox/flox^; CDX2P-CreER^T2^ (–TAM/+TAM) mice on day 28 were measured and presented as mean (n = 3 mice per group, 30 crypts per proximal/distal colon) ± SD, each data point represents an individual mouse. (*B*) Lengths of K8+ and K8– crypts from proximal and distal colon of K8^flox/flox^; CDX2P-CreER^T2^ +TAM mice were quantified and presented as mean (n = 3 mice, 15 K8+ and 15 K8– crypts per proximal/distal colon) ± SD, each data point represents an individual crypt. (*C*) Immunofluorescence staining of K8 (*magenta*), Ki67 (*green*), nuclei, DAPI (*blue*) in proximal and distal colon sections of K8^flox/flox^; CDX2P-CreER^T2^ (–TAM/+TAM) mice (n = 3 mice per group) on day 28 is shown. Areas within the *white dashed lines* represent K8-negative colon crypts; scale bar = 50 μm. All the images are representative of n = 3 mice per group. (*D*) Percentage of Ki67+ cells was quantified in proximal and distal colon of K8^flox/flox^; CDX2P-CreER^T2^ (–TAM/+TAM) mice and presented as mean (n = 3 mice per group, 3 images per proximal/distal colon) ± SD, each data point represents an individual mouse. (*E*) Percentage of Ki67+ cells in K8+ and K8– crypts of proximal and distal colon was calculated in K8^flox/flox^; CDX2P-CreER^T2^ +TAM mice and presented as mean (n = 3 mice, 6–15 K8+ and 8–15 K8– crypts per proximal/distal colon) ± SD, each data point represents an individual crypt. The statistical significance was determined after performing unpaired Student’s *t*-test for (*A* and *B*) and (*D* and *E*), shown as ∗*P* < .05; ∗∗*P* < .01; ∗∗∗*P* < .001; and ∗∗∗∗*P* < .0001.

The differences in crypt length between experimental groups, as well as between crypts with and without K8 expression in TAM-treated K8^flox/flox^; CDX2P-CreER^T2^ mice, were analyzed. In TAM-treated mice, crypt lengths in the proximal colon were significantly increased compared with vehicle-treated controls. In contrast, no statistically significant differences were observed in crypt lengths in the distal colon, with inter-mouse variations compared with vehicle-treated mice (214.2 ± 39.9 vs 175.0 ± 3.4 μm, respectively; *P* = .24) (Figure 7*A*). Within TAM-treated mice, crypts lacking K8 in the proximal and distal colon showed no difference in length compared with crypts retaining K8 expression (Figure 7*B*). To assess the proliferative activity of colonocytes in these mice, the percentage of Ki67+ cells was quantified. Overall, both the proximal and distal colons of TAM-treated mice exhibited a higher percentage of Ki67+ cells compared with vehicle-treated controls (Figure 7*C* and *D*). Notably, K8-negative crypts contained a significantly higher percentage of Ki67+ cells in both the proximal and distal colon compared with K8-positive crypts in TAM-treated K8^flox/flox^; CDX2P-CreER^T2^ mice (Figure 7*E*).

Interestingly, K8-positive crypts in the proximal colon of TAM-treated mice were significantly taller and contained more Ki67+ cells compared with crypts from vehicle-treated mice with intact K8 (Figure 4*B* and *C*). In the distal colon, these K8-positive crypts were on average slightly longer (217.8 ± 31.2 vs 175 ± 3.4 μm, respectively; *P* = .13) and had modestly more Ki67+ cells (12 ± 0.8 vs 7.9 ± 2.5 %, respectively; *P* = .092), however without reaching statistical significance, in comparison to vehicle control crypts (Figure 4*B* and *C*). These data suggest that K8 loss induced pro-proliferative processes may be influenced not only by K8 in local crypts, but also by interactions with neighboring K8-negative crypts and their microenvironment. Together these findings show that the loss of K8 is associated with increased colonocyte proliferation in crypts.

**Figure 8:**
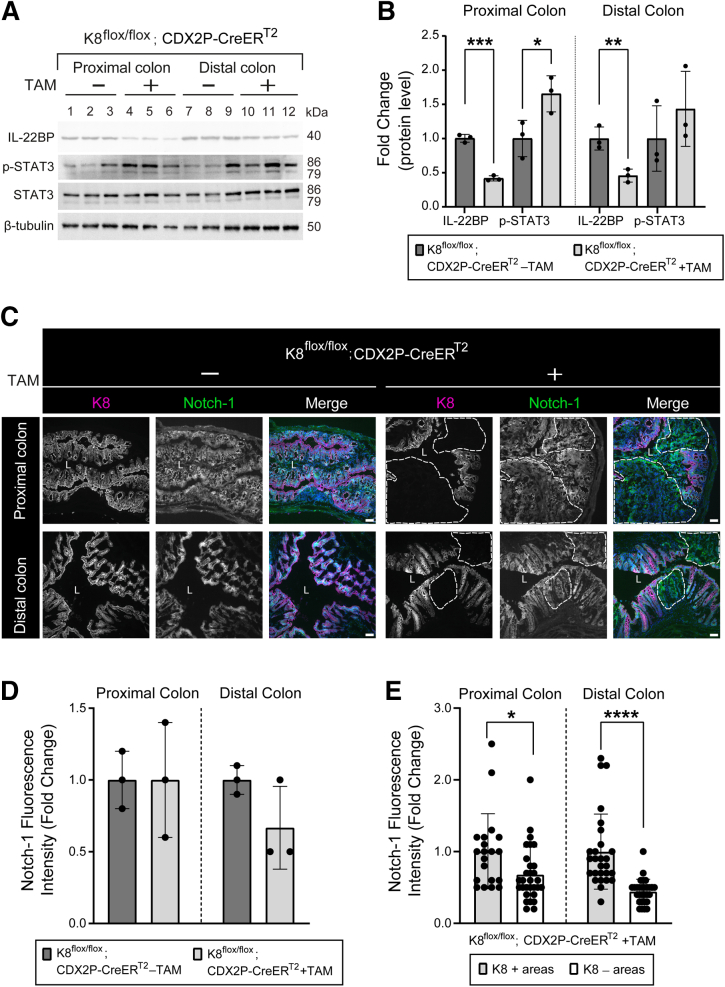
**TAM-treated K8^flox/flox^; CDX2P-CreER^T2^ mice show decreased IL-22BP and increased p-STAT3 levels with reduced Notch-1 expression in the colon.** (*A*) Total proximal colon lysates from K8^flox/flox^; CDX2P-CreER^T2^ –TAM (Lanes 1–3), K8^flox/flox^; CDX2P-CreER^T2^ +TAM (Lanes 4–6) and total distal colon lysates from K8^flox/flox^; CDX2P-CreER^T2^ –TAM (Lanes 7–9), K8^flox/flox^; CDX2P-CreER^T2^ +TAM (Lanes 10–12) on day 28 were immunoblotted for IL-22BP, p-STAT3, and STAT3. β-tubulin was used as the loading control. (*B*) Western blots from (*A*) were quantified and normalized to β-tubulin. The results are presented as mean (n = 3 mice per group, each data point represents an individual mouse) protein fold changes ± SD. (*C*) Immunofluorescence staining of K8 (*magenta*), Notch-1 (*green*), nuclei, DAPI (*blue*) in proximal and distal colon sections of K8^flox/flox^; CDX2P-CreER^T2^ (–TAM/+TAM) mice (n = 3 mice per group) on day 28 is shown. Areas within the *white dashed lines* represent K8-negative colon crypts; scale bar = 50 μm. All the images are representative of n = 3 mice per group. (*D*) Mean fluorescence intensity for Notch-1 was quantified in proximal and distal colon of K8^flox/flox^; CDX2P-CreER^T2^ (–TAM/+TAM) mice and presented as mean fold change (n = 3 mice per group, 2 images per proximal/distal colon) ± SD, each data point represents an individual mouse. (*E*) Mean fluorescence intensity for Notch-1 in K8+ and K8– crypts of proximal and distal colon was measured in K8^flox/flox^; CDX2P-CreER^T2^ +TAM mice and presented as mean fold change (n = 3 mice, 3–10 K8+ and 9–10 K8– crypts per proximal/distal colon) ± SD, each data point represents an individual crypt. The statistical significance was determined after performing unpaired Student’s *t*-test for (*B*, *D*, and *E*), shown as ∗*P* < .05; ∗∗*P* < .01; ∗∗∗*P* < .001; and ∗∗∗∗*P* < .0001.

We next analyzed protein levels of interleukin-22 binding protein (IL-22BP) and phosphorylated signal transducer and activator of transcription 3 (p-STAT3) to investigate STAT3 activation in the colons of these mice. IL-22BP expression is typically downregulated during intestinal damage, which increases IL-22 bioavailability to support epithelial proliferation and tissue repair.^17^ STAT3, an inducer of cell survival and proliferation, is activated as the downstream signaling effect of IL-22, and consistent STAT3 activation exerts a prolonged anti-apoptotic and pro-tumorigenic effect.^18^, ^19^, ^20^ Following colon-specific K8 downregulation in TAM-treated K8^flox/flox^; CDX2P-CreER^T2^ mice, IL-22BP protein levels were significantly decreased in the proximal and distal colon in comparison to vehicle-treated mice with K8. Levels of p-STAT3 were significantly higher in the proximal colon of TAM-treated mice. However, in the distal colon, it did not reach statistical significance despite showing an increased average compared with vehicle-treated mice (1.43 ± 0.44 vs 1 ± 0.39, respectively; *P* = .361). This can be due to the overall more modest K8 loss in distal colon, and lysate samples contain both K8-positive and K8-negative areas (Figure 8*A* and *B*).

Next we, investigated whether Notch-1 expression differed between K8-negative and K8-positive crypts, as Notch-1 is a key regulator of colon epithelial cell differentiation and has been previously shown to interact with K8, influencing its role in epithelial differentiation.^15^ On average, Notch-1 expression did not change significantly between TAM-treated K8^flox/flox^; CDX2P-CreER^T2^ and vehicle-treated mice (Figure 8*C* and *D*). However, within TAM-treated mice, K8-negative crypts in both the proximal and distal colon exhibited reduced Notch-1 expression compared with K8-positive crypts (Figure 8*E*). These findings altogether suggest that with the loss of K8, colon proliferation and differentiation is perturbed, and K8 is essential in maintaining the balance in normal colonocyte proliferation and differentiation.

### K8-negative Areas in the Distal Colon of Tamoxifen-treated K8^flox/flox^; CDX2P-CreER^T2^ Mice Show More Beta-catenin Expression at the Apical Membrane

**Figure 9:**
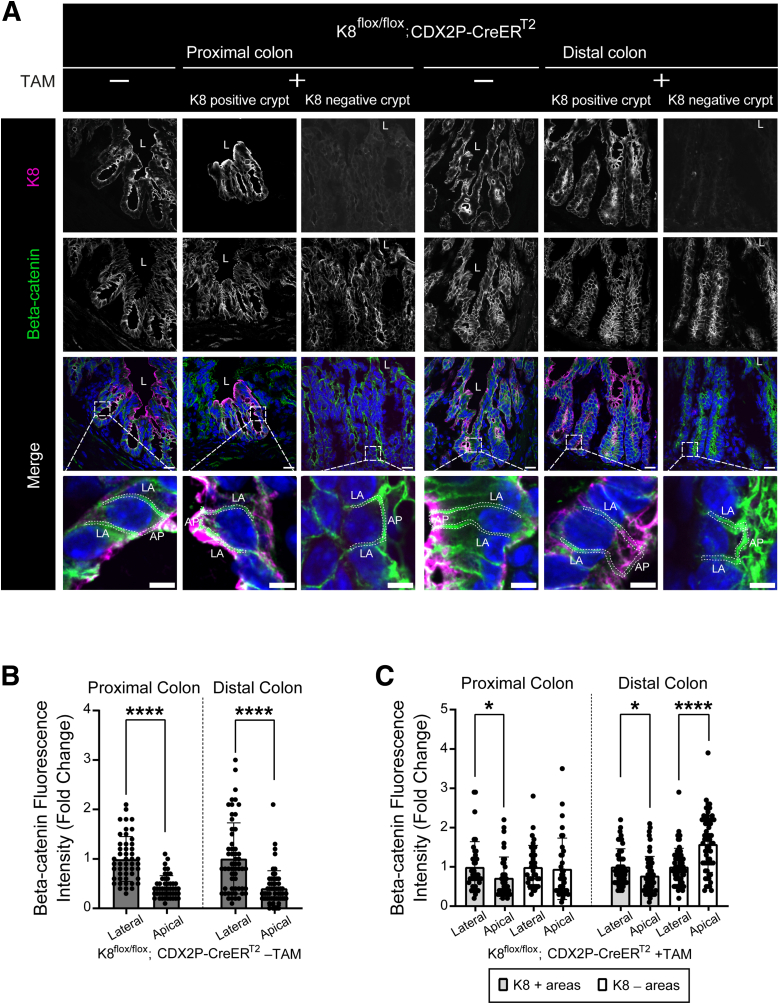
**Distal colon epithelial cells in the K8-negative areas of TAM-treated K8^flox/flox^; CDX2P-CreER^T2^ mice accumulate more beta-catenin at the apical membrane.** (*A*) Immunofluorescence staining of K8 (*magenta*), beta-catenin (*green*), nuclei, DAPI (*blue*) in proximal and distal colon sections of K8^flox/flox^; CDX2P-CreER^T2^ (–TAM/+TAM) mice (n = 3 mice per group) on day 28 is shown; scale bar = 20 μm. In merged images, areas within the *dashed squares* are shown zoomed in the panel below. LA indicates lateral membrane, AP indicates apical membrane, and examples of the regions of interest are shown as *thin dashed lines*; scale bar = 5 μm. All the images are representative of n = 3 mice per group. (*B*) Mean fluorescence intensity for lateral and apical beta-catenin in colon epithelial cells of proximal and distal colon crypts was measured in K8^flox/flox^; CDX2P-CreER^T2^ –TAM mice and presented as mean fold change (n = 3 mice, 2–5 cells in 5 crypts per proximal/distal colon) ± SD, each data point represents an individual cell. (*C*) Mean fluorescence intensity for lateral and apical beta-catenin in colon epithelial cells of proximal and distal colon crypts (K8+ and K8– crypts) was measured in K8^flox/flox^; CDX2P-CreER^T2^ +TAM mice and presented as mean fold change (n = 3 mice, 2–5 cells in 5 K8+ and 5 K8– crypts per proximal/distal colon) ± SD, each data point represents an individual cell. The statistical significance was determined after performing unpaired Student’s *t*-test for (*B* and *C*), shown as ∗*P* < .05; ∗∗*P* < .01; ∗∗∗*P* < .001; and ∗∗∗∗*P* < .0001.

Beta-catenin signaling was examined next, as Wnt/beta-catenin signaling is crucial for the physiological proliferation and differentiation of colon epithelial cells.^21^ In healthy colon epithelial cells, beta-catenin is mainly located at the lateral membrane in the adherens junction complex with E-cadherin in order to mediate cell adhesion.^22^, ^23^, ^24^ Without this lateral clustering of cadherin-catenin complex, cells lose their intercellular adhesion, show impaired apical-basolateral polarity, and affect multiple cellular processes.^25^^,^^26^ In the vehicle-treated mice, colon epithelial cells showed lateral clustering of beta-catenin, as its expression was significantly higher on the lateral side than the apical side of the membrane throughout the colon (Figure 9*A* and *B*). In TAM-treated mice, K8-positive crypts also showed more beta-catenin at the lateral side in both proximal and distal colon. Interestingly, K8-negative crypts only in the distal colon had significantly increased beta-catenin at the apical side compared with the lateral side of the membrane (Figure 9*A* and *C*). This suggests that K8 is required for proper beta-catenin localization in the distal colon epithelial cells to support the cell adhesion and facilitate the apical-basolateral polarity.

### Tamoxifen-treated K8^flox/flox^; CDX2P-CreER^T2^ Mice Reveal Increased Number of Nuclear SRY-box Transcription Factor 9+ Stem and Progenitor Cells in the Distal Colon

**Figure 10:**
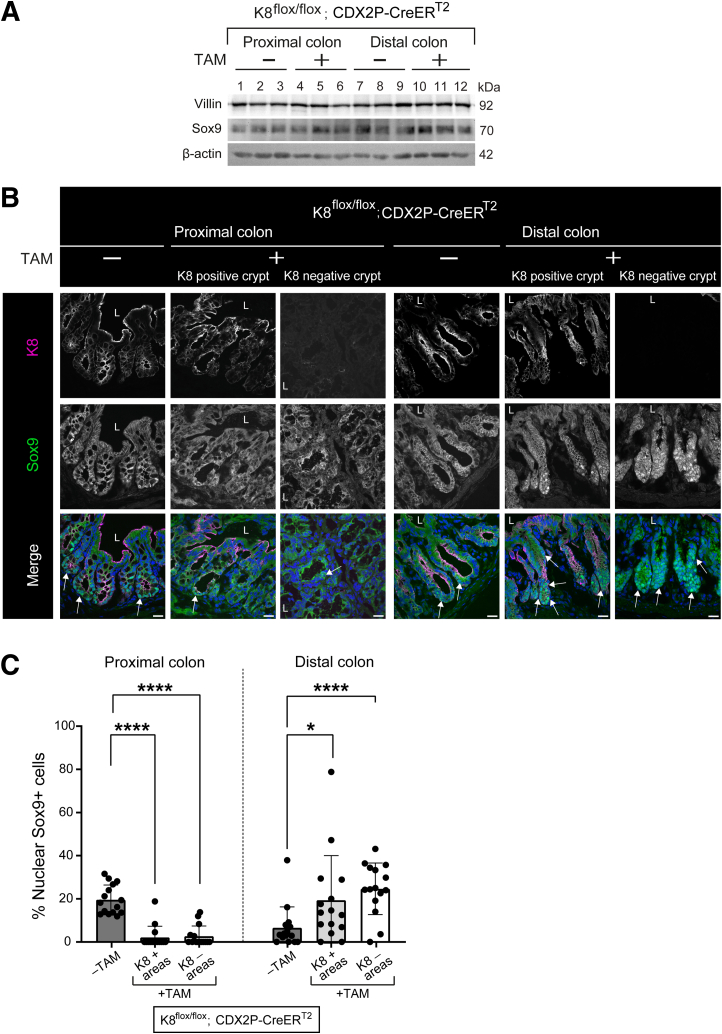
**Distal colon of the TAM-treated K8^flox/flox^; CDX2P-CreER^T2^ mice show higher percentage of nuclear Sox9+ cells.** (*A*) Total proximal colon lysates from K8^flox/flox^; CDX2P-CreER^T2^ –TAM (Lanes 1–3), K8^flox/flox^; CDX2P-CreER^T2^ +TAM (Lanes 4–6) and total distal colon lysates from K8^flox/flox^; CDX2P-CreER^T2^ –TAM (Lanes 7–9), K8^flox/flox^; CDX2P-CreER^T2^ +TAM (Lanes 10–12) on day 28 were immunoblotted for villin and Sox9. β-actin was used as the loading control. (*B*) Immunofluorescence staining of K8 (*magenta*), Sox9 (*green*), nuclei, DAPI (*blue*) in proximal and distal colon sections of K8^flox/flox^; CDX2P-CreER^T2^ (–TAM/+TAM) mice (n = 3 mice per group) on day 28 is shown; *white arrows* indicate nuclear Sox9+ cells; scale bar = 20 μm. All the images are representative of n = 3 mice per group. (*C*) Percentage of nuclear Sox9+ cells was quantified in proximal and distal colon crypts of K8^flox/flox^; CDX2P-CreER^T2^ (–TAM/+TAM) mice and presented as mean (n = 3 mice per group, 5 –TAM, 5 K8+ and 5 K8– crypts per proximal/distal colon) ± SD, each data point represents an individual crypt. The statistical significance was determined after performing unpaired Student’s *t*-test for (*C*), shown as ∗*P* < .05; ∗∗*P* < .01; ∗∗∗*P* < .001; and ∗∗∗∗*P* < .0001.

K8-negative areas of TAM-treated mice displayed enhanced proliferation, consequently the stem, progenitor, and differentiated cell populations were analyzed. The levels of enterocyte protein Villin and stem cell and progenitor cell protein SRY-box transcription factor 9 (Sox9) did not appear to differ between TAM- and vehicle-treated mice in total colon lysates (Figure 10*A*). In colon, Sox9 shows nuclear expression in the stem and progenitor cell compartment at the crypt base.^27^ Sox9 immunofluorescence staining and quantification demonstrated that, in the TAM-treated K8^flox/flox^; CDX2P-CreER^T2^ mice, the percentage of nuclear Sox9+ cells varied throughout the colon (Figure 10*B*). Interestingly, nuclear Sox9+ cells were reduced in the proximal colon compared with vehicle-treated mice, with no difference between K8-positive and K8-negative crypts. In stark contrast, distal colon of TAM-treated mice overall showed higher percentage of nuclear Sox9+ cells than the vehicle-treated mice (Figure 10*C*), with a much stronger increase in K8-negative crypts, whereas there was no statistical difference comparing K8-negative crypts and K8-positive crypts (24.6 ± 2.2 vs 19.3 ± 4.6 %, respectively; *P* =.21) (Figure 10*C*). Altogether, the data suggests that K8 loss leads to variable number of stem and progenitor cells throughout the colon, which further strengthens K8 contribution in maintaining the normal colonocyte proliferation.

### K8-negative Colon of Tamoxifen-treated K8^flox/flox^; CDX2P-CreER^T2^ Mice Have Increased Number of Transit-amplifying Dividing Cells That Show More Asymmetric Division in the Distal Colon

**Figure 11:**
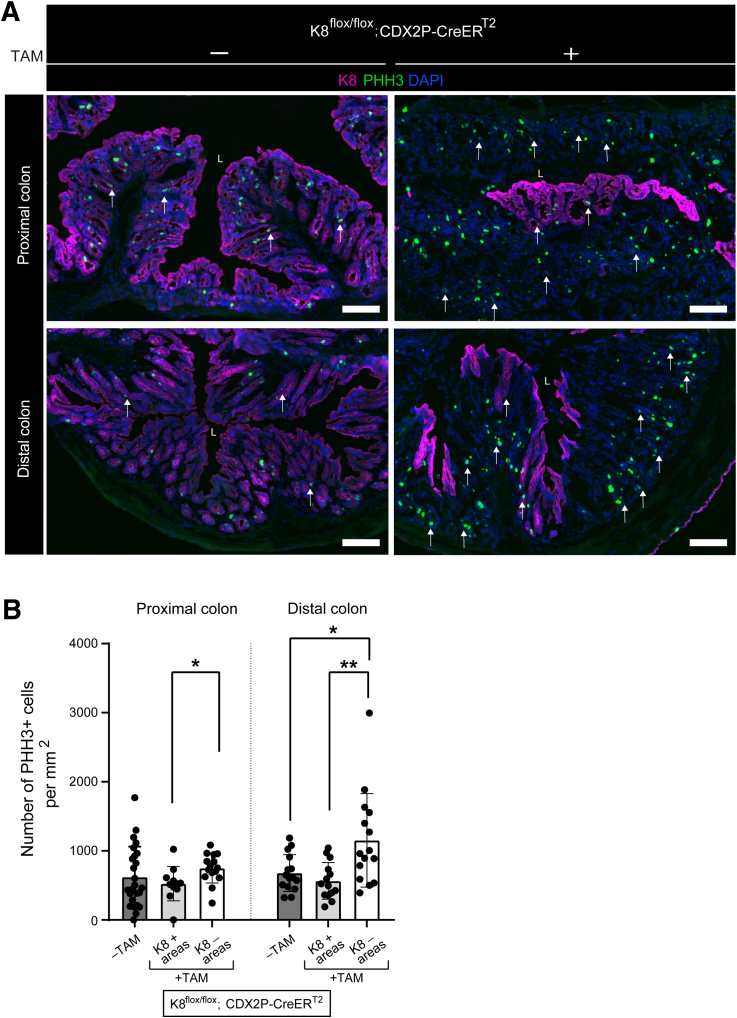
**K8-negative areas of TAM-treated K8^flox/flox^; CDX2P-CreER^T2^ mice colon display increased number of PHH3+ cells.** (*A*) Immunofluorescence staining of K8 (*magenta*), PHH3 (*green*), nuclei, DAPI (*blue*) in proximal and distal colon section of K8^flox/flox^; CDX2P-CreER^T2^ (–TAM/+TAM) mice (n = 3 mice per group) on day 28 is shown; *white arrows* indicate PHH3+ cells; scale bar = 100 μm. All the images are representative of n = 3 mice per group. (*B*) Number of PHH3+ cells was counted in proximal and distal colon crypt areas of K8^flox/flox^; CDX2P-CreER^T2^ (–TAM/+TAM) mice and presented as mean (n = 3 mice per group, 5–8 –TAM, 1–5 K8+ and 5 K8– crypts per proximal/distal colon) ± SD, each data point represents an individual area. The statistical significance was determined after performing unpaired Student’s *t*-test for (*B*), shown as ∗*P* < .05 and ∗∗*P* < .01.

**Figure 12:**
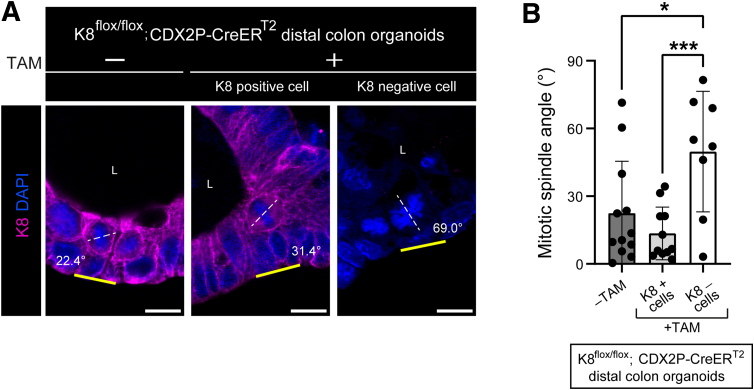
**Distal colon epithelial cells in K8 negative areas of TAM-treated K8^flox/flox^; CDX2P-CreER^T2^ mice organoids divide asymmetrically.** (*A*) Immunofluorescence staining of K8 (*magenta*), nuclei, DAPI (*blue*) in distal colon organoids isolated from untreated K8^flox/flox^; CDX2P-CreER^T2^ mice (n = 2 mice) and thereafter treated (–TAM/+TAM) is shown. *Yellow line* shows basal side of organoid, and *white dashed line* indicates spindle angle, also mentioned in the respective images; scale bar = 10 μm. All the images are representative of n = 9 –TAM and 13 +TAM organoids from 2 mice. (*B*) Spindle orientation was defined by identifying mitotic spindle angle against the basal side of the distal colon organoids and presented as mean (n = 12 –TAM, 11 K8+, and 8 K8– dividing cells) ± SD, each data point represents an individual dividing cell. The statistical significance was determined after performing unpaired Student’s *t*-test for (*B*), shown as ∗*P* < .05; ∗∗*P* < .01; and ∗∗∗*P* < .001.

We next assessed the number of transit amplifying cells with a phosphohistone H3 (PHH3) mitosis marker^28^ by immunofluorescence staining (Figure 11*A*). Overall, the number of PHH3+ cells per crypt area were significantly higher in the K8-negative areas than the K8-positive areas throughout the colon of TAM-treated mice, with a 2-fold increase in K8-negative areas of the distal colon (Figure 11*A* and *B*). Next, to assess the cell division symmetry in the distal colon, dividing cells in K8^flox/flox^; CDX2P-CreER^T2^ distal colon organoids were examined for their spindle pole orientation (Figure 12*A*). K8-negative dividing cells showed more asymmetric cell division than K8-positive cells and vehicle-treated dividing cells, which divided symmetrically (Figure 12*A* and *B*). Taken together, K8 loss markedly increases the number of dividing PHH3+ cells in the distal colon with more asymmetric cell division. In addition to maintaining the number of colonocyte population K8 is involved in deciding the symmetry of colonocyte division.

### Colonic K8 Downregulation Combined With Monoallelic Apc Inactivation Renders K8^flox/flox^; CDX2P-CreER^T2^; Apc^flox/+^ Mice More Susceptible to Colon Tumorigenesis

**Figure 13:**
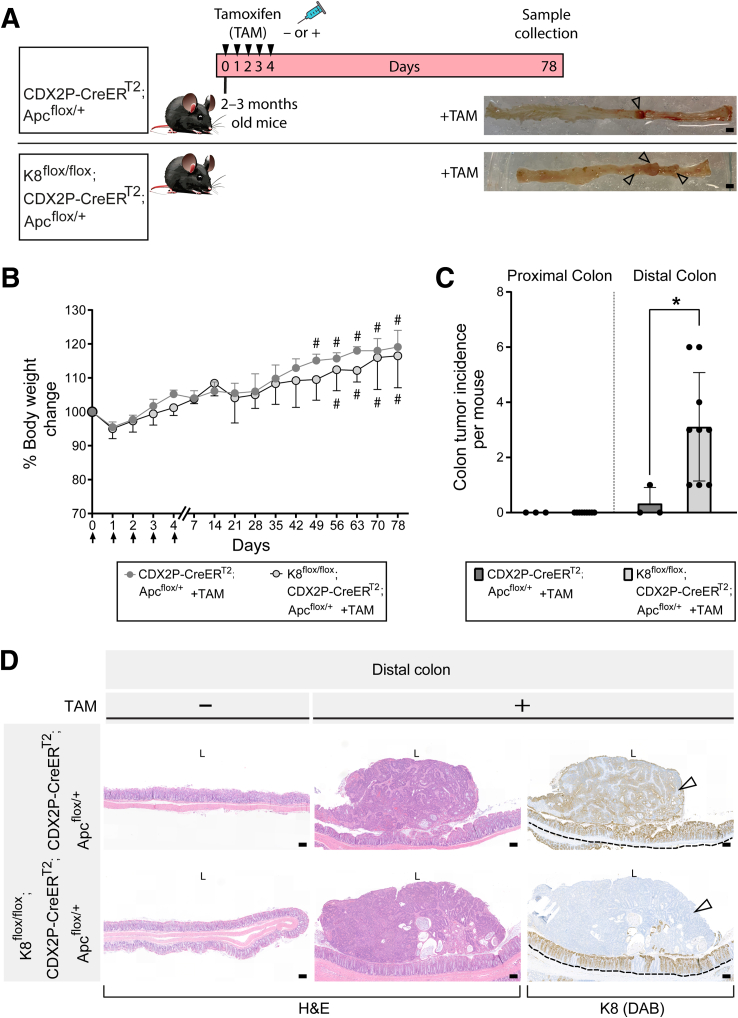
**K8 downregulation in TAM-induced K8^flox/flox^; CDX2P-CreER^T2^; Apc^flox/+^ mice enhances tumor susceptibility in the distal colon.** (*A*) Experimental timeline for CDX2P-CreER^T2^; Apc^flox/+^ and K8^flox/flox^; CDX2P-CreER^T2^; Apc^flox/+^ mice CRC susceptibility study. Adult 2- to 3-month-old mice were administered vehicle (–TAM) or tamoxifen (+TAM), indicated by *black arrowheads*. Open colon images on day 78 with *black arrowheads* pointing toward tumors; scale bar = 5 mm. (*B*) Percentage body weight changes of CDX2P-CreER^T2^; Apc^flox/+^ +TAM and K8^flox/flox^; CDX2P-CreER^T2^; Apc^flox/+^ +TAM mice was determined, and results are shown as mean (n = 3–4 mice per group) ± SD at different time points during the experimental study; *black arrows* indicate –TAM/+TAM administrations, and *hashtags* represent onset of rectal bleeding. (*C*) Number of colonic tumors observed in CDX2P-CreER^T2^; Apc^flox/+^ +TAM (n = 3) and K8^flox/flox^; CDX2P-CreER^T2^; Apc^flox/+^ +TAM (n = 9), results are presented as mean ± SD, each data point represents an individual mouse. CDX2P-CreER^T2^; Apc^flox/+^ (n = 3) and K8^flox/flox^; CDX2P-CreER^T2^; Apc^flox/+^ (n = 9) had their own –TAM controls. (*D*) H&E sections of distal colon from CDX2P-CreER^T2^; Apc^flox/+^ (–TAM/+TAM) and K8^flox/flox^; CDX2P-CreER^T2^; Apc^flox/+^ (–TAM/+TAM) and K8 (DAB) immunolabeling of colon tumors with adjacent colon. For CDX2P-CreER^T2^; Apc^flox/+^ +TAM colon tumor; *black arrowhead* indicates less K8 expression when compared with nearby colon tissue indicated with the *black dashed line* and for K8^flox/flox^; CDX2P-CreER^T2^; Apc^flox/+^ +TAM colon tumor, *black arrowhead* indicates negative K8 expression, with patchy K8 distribution in nearby colon tissue shown with the *black dashed line*; scale bar = 100 μm. The statistical significance was determined after performing 2-way ANOVA with Bonferroni’s post hoc test for (*B*) and unpaired Student’s *t*-test for (*C*), shown as ∗*P* < .05.

**Figure 14:**
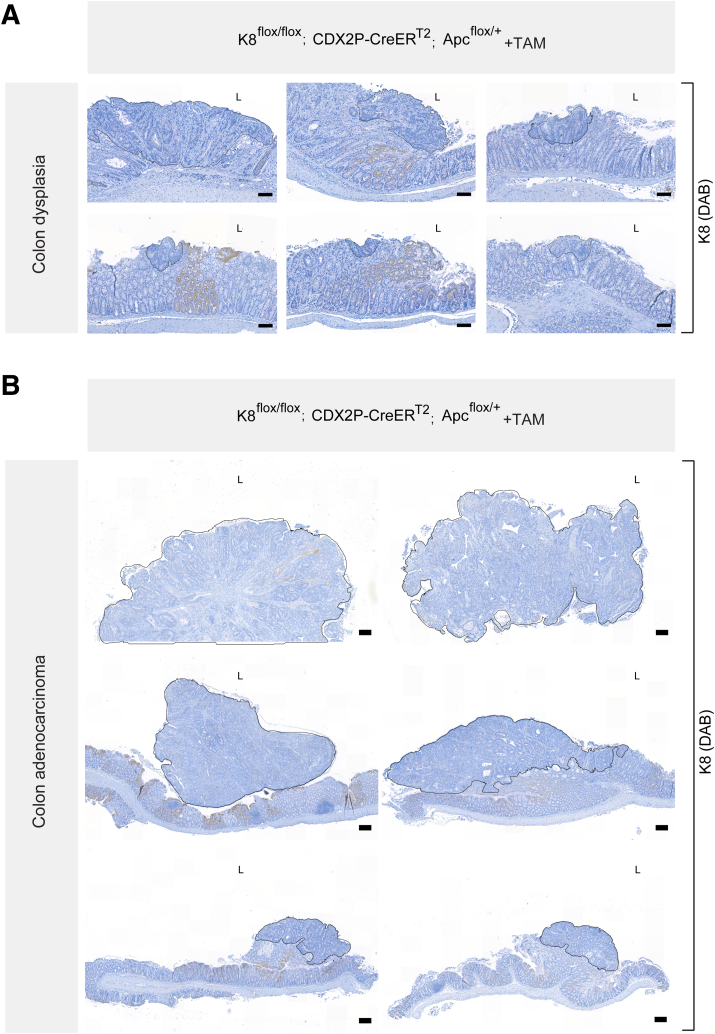
**Dysplastic areas and colon adenocarcinoma in the distal colon of TAM-treated K8^flox/flox^; CDX2P-CreER^T2^; Apc^flox/+^ mice depict negligible K8 expression.** (*A* and *B*) K8 (DAB) immunolabeling in distal colon of K8^flox/flox^; CDX2P-CreER^T2^; Apc^flox/+^ mice, *black encircled* area in (*A*) represents dysplastic growth regions in distal colon; scale bar = 100 μm, and in (*B*) represents colon adenocarcinoma; scale bar = 200 μm. < 1 % of all cells in these *black encircled* areas were K8-positive.

**Figure 15:**
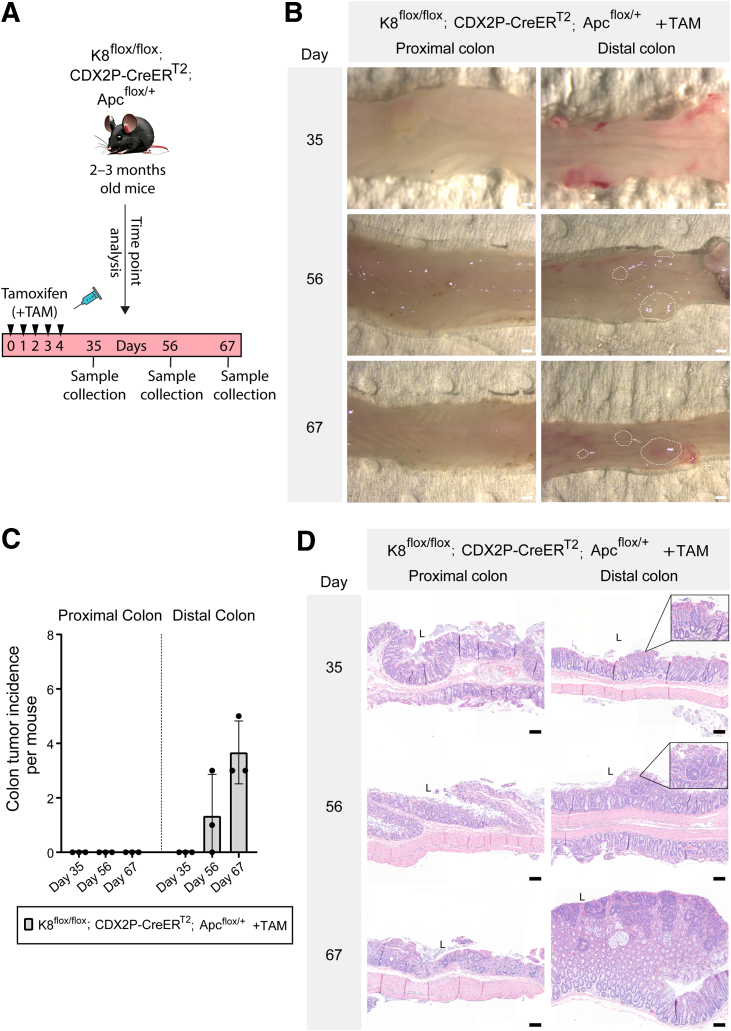
**Abnormal crypt formation begins on day 35 in TAM-treated K8^flox/flox^; CDX2P-CreER^T2^; Apc^flox/+^ mice.** (*A*) Schematic representation of time course analysis for K8^flox/flox^; CDX2P-CreER^T2^; Apc^flox/+^ mouse model. Adult 2- to 3-month-old mice were administered tamoxifen (+TAM), indicated by *black arrowheads*. Samples were collected on days 35, 56, and 67. (*B*) Open colon images on days 35, 56, and 67 with *white dashed* areas representing tumors; scale bar = 10 mm. (*C*) Number of colonic tumors observed in K8^flox/flox^; CDX2P-CreER^T2^; Apc^flox/+^ +TAM (n = 3 mice per day), results are presented as mean ± SD; each data point represents an individual mouse. (*D*) H&E sections of proximal and distal colon from K8^flox/flox^; CDX2P-CreER^T2^; Apc^flox/+^ +TAM mice; scale bar = 100 μm. All the images are representative of n = 3 mice per day.

**Figure 16:**
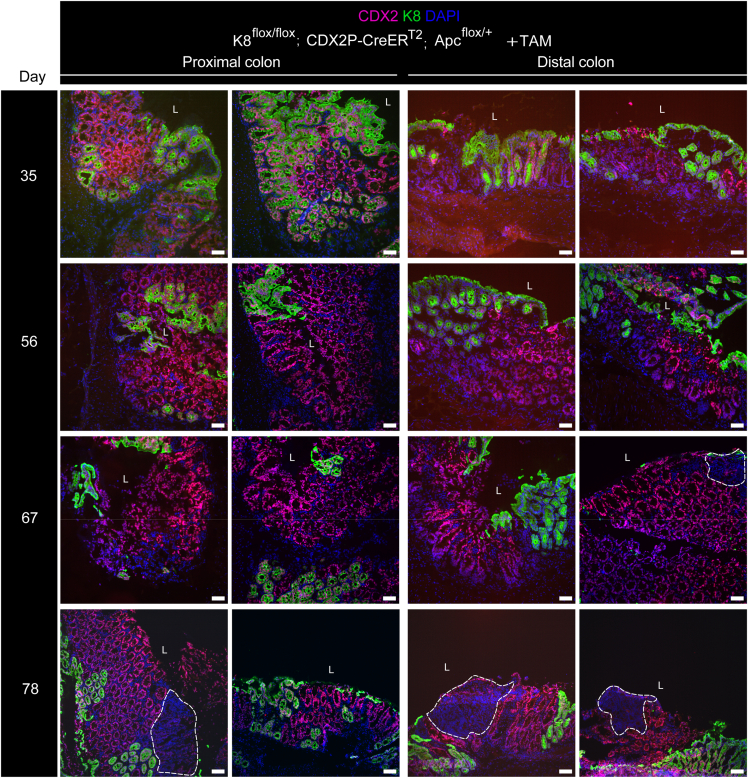
**Colon dysplastic regions and colon adenocarcinoma in TAM-treated K8^flox/flox^; CDX2P-CreER^T2^; Apc^flox/+^ mice exhibit minimal CDX2 expression.** Immunofluorescence staining of K8 (*green*), CDX2 (*magenta*), and nuclei, DAPI (*blue*) in proximal and distal colon sections of K8^flox/flox^; CDX2P-CreER^T2^; Apc^flox/+^+TAM mice (n = 3 mice per day 35, 56, and 67; n = 2 mice per day 78) is shown. Areas within the *white dashed lines* represent colon dysplastic and colon adenocarcinoma regions. For days 35, 56, and 67, scale bar = 50 μm and for day 78, scale bar = 100 μm. All the images are representative of n = 2–3 mice per day.

**Figure 17:**
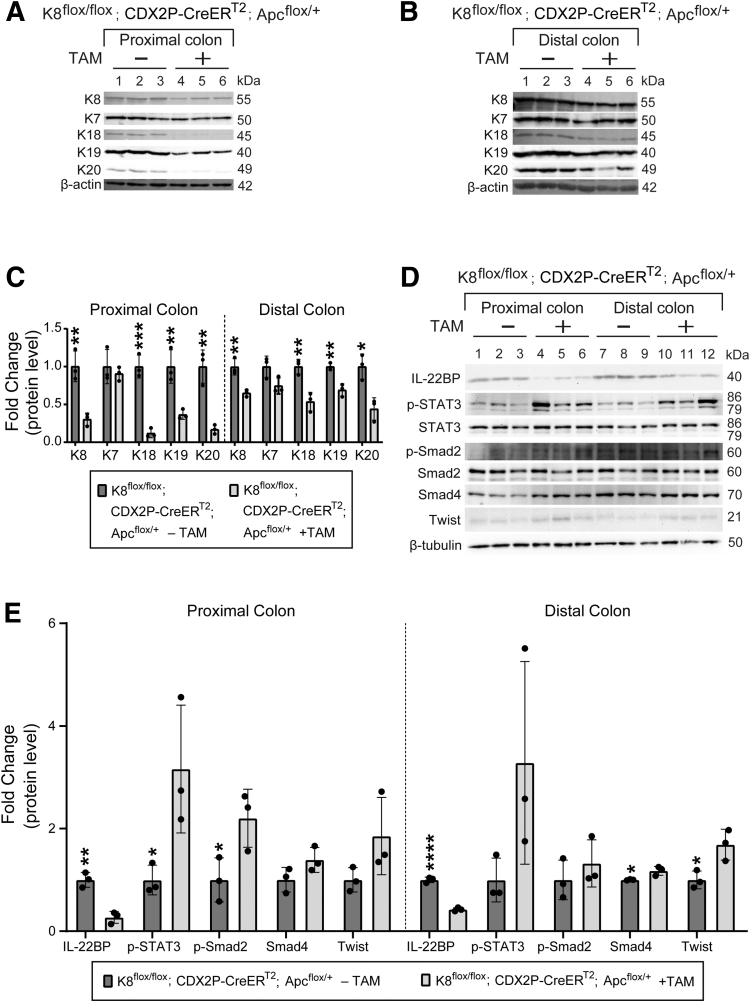
**TAM-treated K8^flox/flox^; CDX2P-CreER^T2^; Apc^flox/+^ mice show downregulation of colonic keratins and minor changes in EMT-associated signaling in the colon.** (*A*) Total proximal colon lysates from K8^flox/flox^; CDX2P-CreER^T2^; Apc^flox/+^ –TAM (Lanes 1–3), K8^flox/flox^; CDX2P-CreER^T2^; Apc^flox/+^ +TAM (Lanes 4–6) and (*B*) total distal colon lysates from K8^flox/flox^; CDX2P-CreER^T2^; Apc^flox/+^ –TAM (Lanes 1–3), K8^flox/flox^; CDX2P-CreER^T2^; Apc^flox/+^ +TAM (Lanes 4–6) on day 78 were immunoblotted for K8, K7, K18, K19, and K20. β-actin was used as the loading control. (*C*) Western blots from (*A* and *B*) were quantified and normalized to β-actin. The results are presented as mean (n = 3 mice per group, each data point represents an individual mouse) protein fold changes ± SD. (*D*) Total proximal colon lysates from K8^flox/flox^; CDX2P-CreER^T2^; Apc^flox/+^ –TAM (Lanes 1–3), K8^flox/flox^; CDX2P-CreER^T2^; Apc^flox/+^ +TAM (Lanes 4–6) and total distal colon lysates from K8^flox/flox^; CDX2P-CreER^T2^; Apc^flox/+^ –TAM (Lanes 7–9), K8^flox/flox^; CDX2P-CreER^T2^; Apc^flox/+^ +TAM (Lanes 10–12) on day 78 were immunoblotted for IL-22BP, p-STAT3, STAT3, p-Smad2, Smad2, Smad4, and Twist. β-tubulin was used as the loading control. (*E*) Western blots from (*D*) were quantified and normalized to β-tubulin. The results are presented as mean (n = 3 mice per group, each data point represents an individual mouse) protein fold changes ± SD. The statistical significance was determined after performing unpaired Student’s *t*-test for (*C and E*), shown as ∗*P* < .05; ∗∗*P* < .01; ∗∗∗*P* < .001; and ∗∗∗∗*P* < .0001.

To determine whether colonocyte K8 downregulation combined with Apc inactivation promotes colon tumor development, CDX2P-CreER^T2^; Apc^flox/+^ and K8^flox/flox^; CDX2P-CreER^T2^; Apc^flox/+^ mice were treated with tamoxifen intraperitoneally for 5 consecutive days and followed for 78 days (Figure 13*A*). The percentage of body weight change between TAM-treated CDX2P-CreER^T2^; Apc^flox/+^ mice and K8^flox/flox^; CDX2P-CreER^T2^; Apc^flox/+^ mice showed no difference over the course of the experiment. One TAM-treated CDX2P-CreER^T2^; Apc^flox/+^ (which had developed 1 colon tumor) and 1 of the TAM-treated K8^flox/flox^; CDX2P-CreER^T2^; Apc^flox/+^ mouse began to show rectal bleeding on day 49 and day 56, respectively (Figure 13*B*). Only 1 of 3 TAM-treated CDX2P-CreER^T2^; Apc^flox/+^ mice developed a single tumor in the distal colon, indicating that the model works. In contrast, all TAM-treated K8^flox/flox^; CDX2P-CreER^T2^; Apc^flox/+^ mice had multiple tumors in the distal colon (Figure 13*C*). To evaluate whether these tumors originated from K8-negative areas, all tumors from both groups were analyzed for K8 expression. The single tumor in TAM-treated CDX2P-CreER^T2^; Apc^flox/+^ mice showed decreased K8 expression compared with surrounding normal colon epithelium. Conversely, all dysplastic lesions and adenocarcinomas in TAM-treated K8^flox/flox^; CDX2P-CreER^T2^; Apc^flox/+^ mice were K8-negative with patchy K8 distribution in the nearby colonic epithelium (Figure 13*D*; Figure 14*A* and *B*). To define the timeline of colon tumorigenesis, TAM-treated K8^flox/flox^; CDX2P-CreER^T2^; Apc^flox/+^ mice were sacrificed on days 35, 56, and 67 after the first TAM administration (Figure 15*A*). There was no observation of tumors on day 35; however, mice began to show aberrant crypt formation in the distal colon (Figure 15*B* and *D*). Two of 3 mice developed tumors on day 56. All 3 mice, on day 67, developed multiple tumors in the distal colon (Figure 15*B* and *D*), and 2 of 3 mice showed rectal bleeding before day 67. Interestingly, areas with colon dysplasia and colon tumors exhibited minimal expression of CDX2 compared with surrounding colonic tissue (Figure 16). In CRC, loss of CDX2 has been linked with advanced disease stage and epithelial to mesenchymal transition (EMT).^29^^,^^30^ K8 and the other keratins were significantly downregulated in both the proximal and distal colons of TAM-treated K8^flox/flox^; CDX2P-CreER^T2^; Apc^flox/+^ mice compared with vehicle-treated controls on day 78 (Figure 17*A–C*) as expected. These findings demonstrate that K8 downregulation in combination with Apc inactivation dramatically increases the susceptibility towards colon tumorigenesis compared with Apc inactivation alone. The early signs of abnormal crypt foci become apparent after 1 month, and tumors are developed around 2 months.

### Colon Adenocarcinoma Cells of K8^flox/flox^; CDX2P-CreER^T2^; Apc^flox/+^ Mice Show Subtle but Consistent EMT

**Figure 18:**
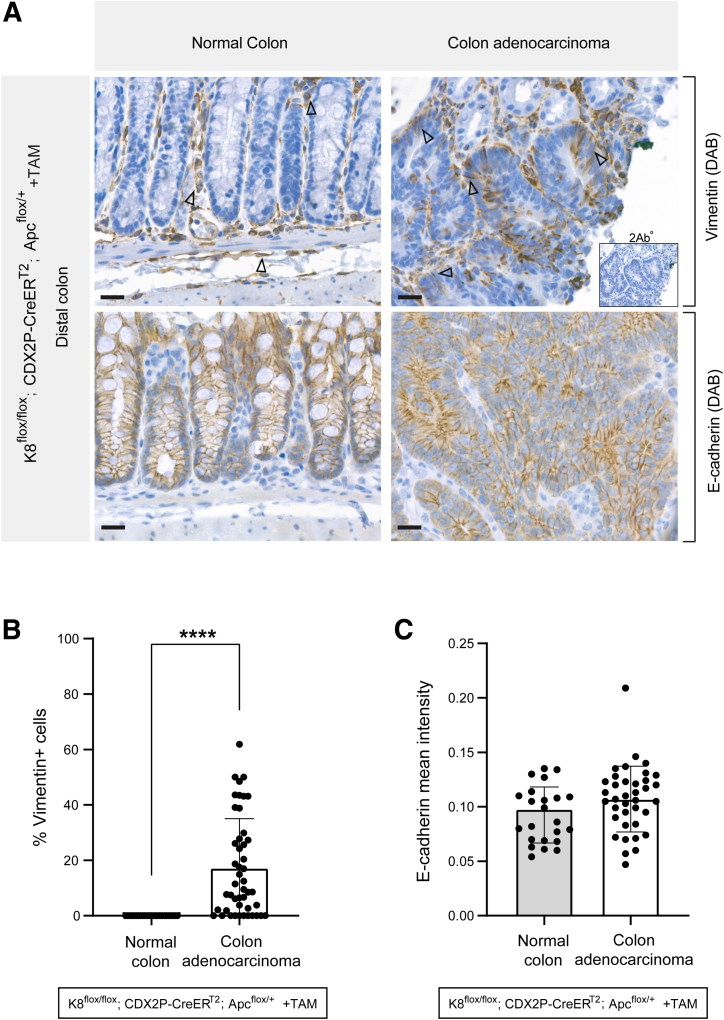
**Colon adenocarcinoma cells in TAM-induced K8^flox/flox^; CDX2P-CreER^T2^; Apc^flox/+^ mice express vimentin.** (*A*) Vimentin and E-cadherin (DAB) immunolabeling in normal colon and colon adenocarcinoma of K8^flox/flox^; CDX2P-CreER^T2^; Apc^flox/+^ (+TAM) mice (n = 3) on day 78, *black arrows* indicate vimentin; scale bar = 50 μm. (*B*) Percentage of vimentin+ cells was measured in the colon epithelial cells of normal colon and colon adenocarcinoma areas and presented as mean (n = 3 mice, 5–10 normal colon and 10–21 colon adenocarcinoma areas per distal colon) ± SD, each data point represents an individual area. (*C*) Mean intensity for E-cadherin was quantified in the normal colon and colon adenocarcinoma areas and presented as mean (n = 3 mice, 5–11 normal colon and 7–23 colon adenocarcinoma areas per distal colon) ± SD, each data point represents an individual area. All the images are representative of n = 3 mice. The statistical significance was determined after performing unpaired Student’s *t*-test for (*B* and *C*), shown as ∗*P* < .05; ∗∗*P* < .01; ∗∗∗*P* < .001; and ∗∗∗∗*P* < .0001.

The increased tumor load and the reduced CDX2 expression in the colon adenocarcinomas in K8- and Apc-deficient mice after 78 days of this model suggested a potential initiation of EMT. Indeed subtle activation of the EMT-associated signaling including STAT3^31^ and transforming growth factor beta-1 (TGF-β1)^32^ were observed in the colon of TAM-treated K8^flox/flox^; CDX2P-CreER^T2^; Apc^flox/+^ mice, and IL-22BP protein levels were similarly reduced. Increased levels of p-STAT3 and p-Smad2 were detected in the proximal colon of TAM-treated compared with vehicle-treated mice. The EMT transcription factor Twist and Smad4 were significantly increased especially in the distal colon (Figure 17*D* and *E*). We next analyzed the mesenchymal marker vimentin expression patterns by immunohistochemistry and identified that the colon adenocarcinoma epithelial cells in TAM-treated mice gained significant vimentin expression. On the contrary, epithelial cells in the normal colon tissue sections of these mice did not express vimentin (Figure 18*A* and *B*). E-cadherin levels were not altered in colon adenocarcinomas (Figure 18*A* and *C*). These results show that the K8 loss induced colon adenocarcinoma development exhibits initial signs of EMT, further highlighting the protective role of K8 in colon tumorigenesis.

### K8 Expression is Downregulated in Human Colon Adenocarcinoma

**Figure 19:**
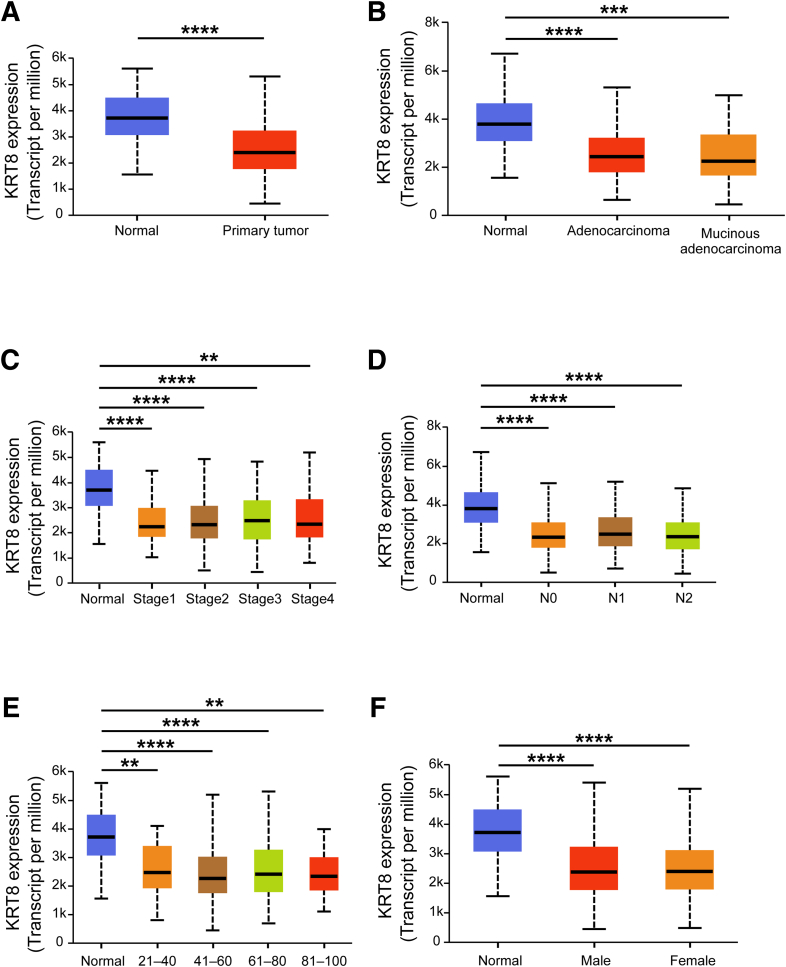
**Transcriptional expression analysis using UALCAN platform reveals downregulated K8 expression in human colon adenocarcinoma in multiple comparisons.** (*A–F*) Comparison of K8 transcriptional expression between (*A*) sample types: normal (n = 41) and primary tumor (n = 286); (*B*) histological subtypes: normal (n = 41), adenocarcinoma (n = 243), and mucinous adenocarcinoma (n = 37); (*C*) individual cancer stages: normal (n = 41), stage 1 (n = 45), stage 2 (n = 110), stage 3 (n = 80), and stage 4 (n = 39); (*D*) nodal metastasis status: normal (n = 41), N0 (n = 166), N1 (n = 70), N2 (n = 47). N0, No regional lymph node metastasis; N1, metastases in 1 to 3 axillary lymph nodes; N2, metastases in 4 to 9 axillary lymph nodes; N3, metastases in 10 or more axillary lymph nodes. (*E*) Patient’s age: normal (n = 41), 21–40 years (n = 12), 41–60 years (n = 90), 61–80 years (n = 149), and 81–100 years (n = 32). (*F*) Patient’s gender: normal (n = 41), male (n = 156), and female (n = 127). The statistical significance shown as ∗*P* < .05; ∗∗*P* < .01; ∗∗∗*P* < .001; and ∗∗∗∗*P* < .0001.

**Figure 20:**
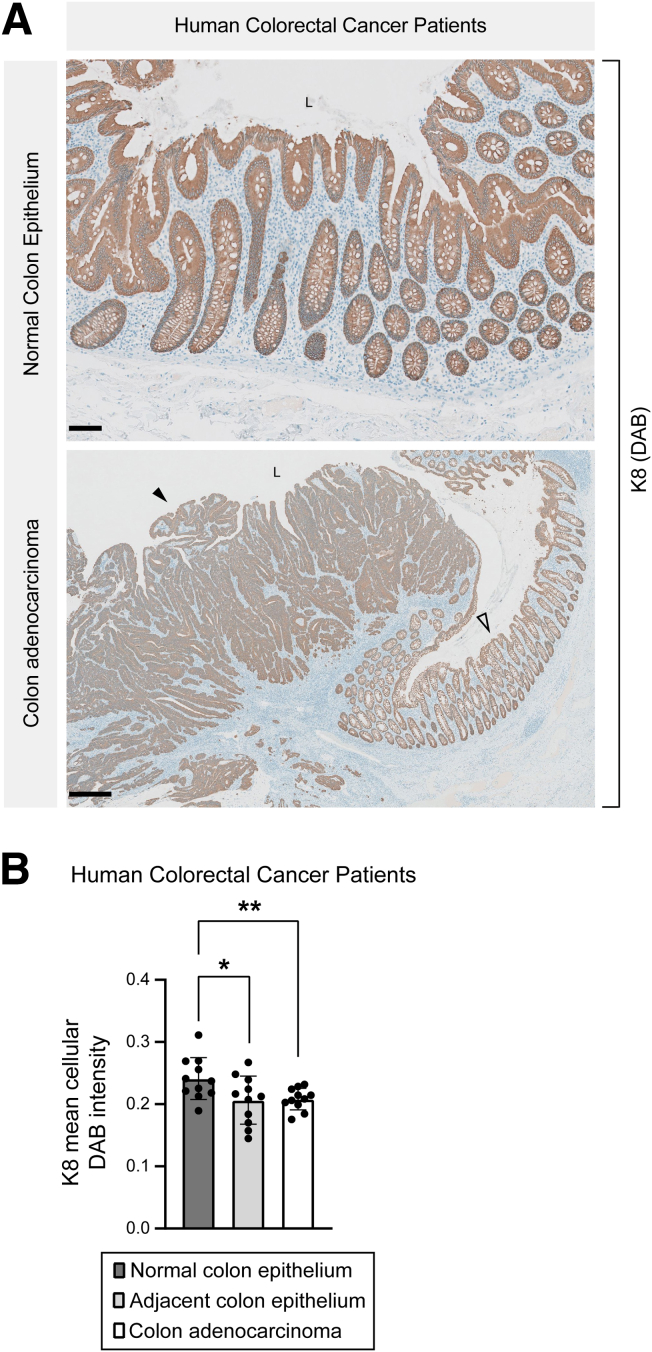
**K8 expression is reduced in colon adenocarcinoma compared with normal colon epithelium in patients with CRC.** (*A*) K8 (DAB) immunolabeling in normal colon epithelium and colon adenocarcinoma with adjacent colon epithelium of patients with CRC, *black filled arrowhead* indicates tumor, and *black unfilled arrowhead* shows adjacent areas; scale bar = 100 μm for normal colon epithelium and scale bar = 500 μm for colon adenocarcinoma. (*B*) Mean cellular DAB intensity for K8 was measured in the normal colon epithelium, colon adenocarcinoma, and adjacent colon epithelium areas and presented as mean (n = 11 patients with CRC, 1 normal colon area [at least 1000 epithelial cells], 1 colon adenocarcinoma area [at least 2000 epithelial cells], and 1 adjacent colon area [at least 1000 epithelial cells] per patient) ± SD, each data point represents an individual area. All the images are representative of n = 11 patients. The statistical significance was determined after performing unpaired Student’s t test for (*B*), shown as ∗*P* < .05 and ∗∗*P* < .01.

To determine whether the correlation between K8 loss and tumorigenesis observed in mice is recapitulated in humans, K8 transcriptional expression levels were analyzed in colon adenocarcinoma and normal colon tissue using The Cancer Genome Atlas Colon Adenocarcinoma (TCGA-COAD) dataset mined in UALCAN. A significant reduction in K8 transcriptional expression was observed in primary tumors from patients with COAD, including adenocarcinoma and mucinous adenocarcinoma, compared with normal colon (Figure 19*A* and *B*). Interestingly, decreased K8 expression was evident at early stages of colon cancer and was independent of metastasis. K8 expression remained reduced in patients with advanced-stage disease, including those with metastasis to 10 or more lymph nodes (Figure 19*C* and *D*). This downregulated K8 expression signature was consistent across both younger and older patients, regardless of gender (Figure 19*E* and *F*). To validate this, K8 expression in the normal colon, adenocarcinoma, and its nearby areas of human patients with CRC was investigated by immunohistochemistry. K8 expression was significantly decreased in the colon adenocarcinoma and its adjacent areas when compared with the normal colon epithelium in the same patients (Figure 20*A* and *B*). Taken together, these findings highlight that K8 is decreased in colon adenocarcinoma. Considering K8 downregulation already at stage1 in UALCAN database suggests that it may represent an early event in the development of colon cancer.

## Discussion

**Figure 21:**
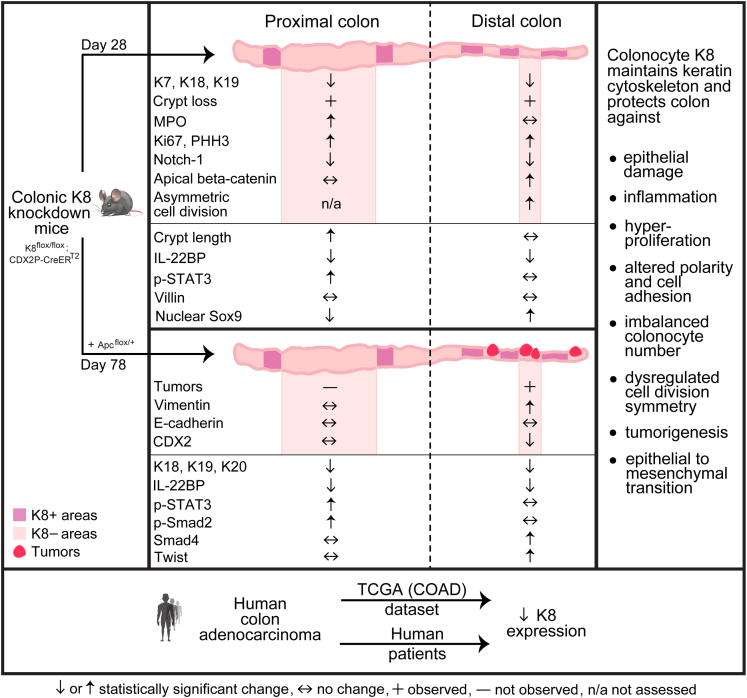
**Graphical summary of molecular and histopathological features of colonic epithelial-specific K8-deletion alone and in combination with monoallelic Apc-deletion for new human CRC like tumorigenesis model.** This graphical summary highlights the molecular features identified in the 2 transgenic inducible knockout mouse models analyzed in this study (K8^flox/flox^; CDX2P-CreER^T2^ and K8^flox/flox^; CDX2P-CreER^T2^; Apc^flox/+^) when colonocyte-specific gene deletion was activated in adult mice with TAM. The molecular and histopathological changes are shown in *white areas* as analyzed in the whole section samples of the proximal or distal colon compared with vehicle control, and in *pink* when analyzed in K8-negative areas compared with K8-positive areas in the TAM-induced mice colon where K8 is deleted in a patchy fashion. *Red* indicates tumors. In summary, distal and proximal colon show similar proliferation signatures; however, in the proximal colon, K8 loss leads to crypt damage and inflammation and is likely essential for tissue repair and regenerative processes, whereas in the distal parts, loss of K8 creates a pro-proliferative environment with increases in progenitor cell compartment, decreased Notch-1 signaling, and asymmetric cell division. The combined loss of K8 and Apc leads to development of distal colon tumors that show signs of EMT. This model resembles humans as K8 levels are decreased in patients with CRC.

In this study, we report the development and phenotypic characterization of a novel mouse model for colon epithelial cell tumorigenesis driven by the combined loss of K8 and Apc (summarized in Figure 21 and the graphical abstract). This genetically induced model does not rely on carcinogens such as AOM, which have variable dose efficacies and long latency periods depending on the mouse strain.^33^ Importantly, K8 levels remained unchanged in the ileum, making the patchy K8 knockout pattern specific to the colon epithelium allowing for colonocyte-autonomous CRC studies. The K8 patchiness mirrors the distribution of K8 expression in human CRC, where undifferentiated tumors show patchy K8 expression, whereas differentiated tumors exhibit stronger expression at the crypt top than at the crypt bottom.^34^ Because mice rapidly developed colon adenocarcinomas already within 2 months following the first TAM dose and exhibiting enhanced colon tumorigenesis compared with mice with Apc loss alone, this new model bears promise for modeling CRC. The model in this study also closely mimics human CRC, which frequently features Apc inactivation and reduced K8 levels. Tumors in this model develop predominantly in the distal colon, consistent with the primary tumor sites in human CRC. Knockout models of keratin-associated proteins (eg, plectin, desmoplakin) that have been developed share several colonic stress and disease-phenotypes with K8 knockout models; however, the early colon tumorigenesis and initiation of EMT in the colon-specific K8-driven model have to our knowledge not been studied in those models in detail.^11^^,^^35^, ^36^, ^37^, ^38^ Nevertheless, these studies emphasize the importance of identifying the K8 interactomics in colonocytes.

Existing Apc-based mouse models (Apc^1638/+^, Apc^Δ716/+^, Apc^Δ14/+^, Apc^Min-FCCC/+^) typically result in a high tumor burden in the small intestine rather than the colon.^6^^,^^39^, ^40^, ^41^, ^42^ Moreover, models such as Apc^Min/+^, Apc^Δ14/+^, Apc^Min-FCCC/+^ also develop tumors in the mammary gland.^41^, ^42^, ^43^ CDX2P-NLS Cre; Apc^+/loxP^ mice have shifted tumor load towards colon, with still some tumors in the small intestine, whereas Villin-Cre; Apc^+/loxP^ mice predominantly develop tumors in the small intestine.^44^ By incorporating TAM-regulated CreER^T2^ under the CDX2P promoter, biallelic Apc inactivation was targeted to the colonic epithelium in adult CDX2P-CreER^T2^; Apc^flox/flox^ mice.^9^ Our model offers a significant advantage over these Apc mutant models, as tumors are restricted to the distal colon. Other multigene-based CRC models, often involve prolonged endpoints, lethal phenotypes, and complex breeding strategies.^45^ These models do not acquire the mutations sequentially but are induced at the same time and in all cells, which is not the case in human CRC.^46^ Most CRC cases (75%–80%) arise as a result of sequential accumulation of somatic gene alterations over time.^47^

The here identified mild colitis-like phenotype in TAM-treated K8^flox/flox^; CDX2P-CreER^T2^ mice with patchy loss of keratins highlights the role of basal keratin levels in maintaining epithelial integrity. Reduced keratin levels have been associated with compromised stress-protective functions, as seen in K8^+/–^ mice, which exhibit moderate colon hyperproliferation and altered ion transport without overt inflammation.^14^^,^^35^^,^^48^^,^^49^ However, heterozygous K8 deletion increases susceptibility to dextran sulfate sodium (DSS)-induced inflammation and AOM/DSS- or Apc^Min/+^/DSS-induced CRC.^14^^,^^35^^,^^49^ The colon epithelial damage and modest inflammation in TAM-treated K8^flox/flox^; CDX2P-CreER^T2^ mice occurred in a K8 expression-dependent manner. Essentially, the crypt loss occurred simultaneously with the appearance of patchy K8 loss as early as day 5 after the first TAM administration. However, modest inflammation occurred only in the proximal colon of these mice. This finding is comparable to full-body K8^–/–^ mice, where inflammation was noticeably higher in the proximal colon than in the distal colon.^50^ Altogether, this highlights an anti-inflammatory role of K8 locally in the proximal colon. One potential cause can be different immune cell populations and immune responses in proximal^51^ and distal colon.^52^, ^53^, ^54^ Notably, pattern recognition receptors (PRRs) including toll-like receptors (TLRs), show variable expression throughout the colon.^55^ TLR9 expression was significantly upregulated only in the proximal colon of full-body K8^–/–^ mice.^50^ Furthermore, K8 has been shown to mitigate the effects of TLR-mediated inflammatory response.^56^

In TAM-treated K8^flox/flox^; CDX2P-CreER^T2^ mice, K8-negative colonic crypts exhibited increased proliferation, decreased IL-22BP, and increased p-STAT3 levels, creating a pro-proliferative environment. This aligns with previous findings linking early K8 loss to the activation these proliferative pathways.^11^^,^^14^^,^^16^ IL-22BP deficiency prolonged epithelial proliferation and increased susceptibility to colon tumorigenesis in AOM/DSS and Apc^Min/+^ models.^17^ In a primary dataset of patients with CRC, IL-22BP was also downregulated.^57^ IL-22 treatment in vivo and in vitro increased intestinal epithelial cell proliferation by inhibiting Notch-1 and Wnt signaling.^58^ Notch-1 expression was also decreased in K8-negative colonic crypts in the current study. These results altogether suggest that the colon epithelial K8 is essential for normal proliferation activity of colonocytes and protects the colonocyte against epithelial hyperproliferation.

Altered beta-catenin cytoplasmic stabilization or nuclear activation was not observed in the TAM-treated K8^flox/flox^; CDX2P-CreER^T2^ mice. However, in the K8-negative crypts beta-catenin was localized more towards the apical membrane in the distal but not proximal colon. This indicates that the K8 loss subtly disrupts cell-cell adhesion and induces loss of polarity in the distal colon, which could contribute to the pro-proliferative phenotype in K8-deficient cells. Beta-catenin is required laterally at adherens junctions to sustain the cell adhesion and polarity.^22^, ^23^, ^24^ Previously, K19 and K5 have been independently shown to interact with beta-catenin in breast cancer cells,^59^^,^^60^ and loss of K19 reduced breast cancer cell adhesion.^61^ K8/K18 binding to Albatross is required to properly form the apical junctional complex (AJC) and maintain epithelial cell polarity.^62^ Keratins are, thus needed to ensure a proper cell polarity in simple epithelia.^63^

Evaluating the progenitor and stem cell compartments, our data shows that the number of these cells varied between the proximal and distal colon in the TAM-treated K8^flox/flox^; CDX2P-CreER^T2^ mice. This was seen by an increase of PHH3-positive transit amplifying cells in K8-negative regions in the distal colon, with a minimal increase in the proximal colon. Similarly, an increased nuclear localization of the stem cell marker Sox9 in K8-negative crypts, compared with a milder effect in K8-positive crypts, was observed in the distal colon, whereas loss of K8 in the proximal colon environment rather decreased the number of cells with nuclear Sox9. Earlier findings reported that colon epithelial cells with high Sox9 expression exhibited stem cell-like properties, whereas the cells with low Sox9 expression demonstrated a more differentiated state.^27^^,^^64^^,^^65^ Increased Sox9 expression was also observed in the biallelic inactivated CDX2P-CreER^T2^; Apc^flox/flox^ mice.^9^ In our study, high nuclear Sox9+ cells were observed in the K8^flox/flox^; CDX2P-CreER^T2^ mice before the second hit with Apc inactivation, highlighting a regulatory function of K8 in maintaining the colonocyte population. CRC cells often opt for asymmetric division, which has a pro-tumorigenic effect^66^ and contributes to intra-tumor heterogeneity.^67^ Aligning with this, K8-negative dividing cells in distal colon derived organoids, divided more asymmetrically, likely enhancing the tumorigenic potential oin distal colon. These data suggest that K8 has differential role in maintaining a balanced stem and progenitor cell population in the distal and proximal colon, and that K8 is involved in deciding the symmetry of dividing cells in the distal colon. Overall, data highlights a major regulatory role of K8 in proliferation primarily in the distal colon as loss of K8 specifically in the distal colon leads to a pro-tumorigenic environment.

Interestingly, despite modest inflammation and proliferation in the proximal colon of our model, no tumors were observed in this region. This may be due to increased crypt damage and colonocyte loss in the proximal colon. Additional to proliferation, more apical beta-catenin, overpopulated stem and progenitor cells and asymmetric cell division in the distal colon contribute primarily to this site-specific tumorigenesis. These events have been associated with colon tumorigenesis.^68^, ^69^, ^70^, ^71^, ^72^ The anatomical tumor distribution makes our model particularly relevant for studying left-sided CRC, which often initiates with Apc inactivation.^73^, ^74^, ^75^ In our model, the precise loss of K8 and Apc accelerates tumorigenesis in the distal colon, consistent with the “just-right” hypothesis, which suggests that Apc inactivation and downstream signaling must occur within an optimal range to drive tumor initiation.^76^^,^^77^ The present study and recent findings^78^ together also highlight the importance of separately analyzing the different regions in the colon for detailed understanding of regional signaling related to tumorigenesis.

In our model, no initiation of metastasis was observed within 78 days. Partial EMT is a highly dynamic and reversible process,^79^ that occurred in the colon adenocarcinoma cells as a modest activation of EMT signaling. Intriguingly, Sox9 overexpression reportedly induced EMT markers^65^ and K8-negative areas in our model expressed higher number of nuclear Sox9+ cells. Additionally, patients with CRC have demonstrated higher Sox9 expression in the tumor compared with the normal colon.^65^ Colon tumors in our model had negligible CDX2 expression, despite its expression in the adjacent colon epithelium and CDX2 loss is associated with more aggressive CRC and increased EMT.^80^ Loss of keratins have been previously implicated in the EMT,^81^ and through other mechanisms than classical EMT.^82^ The here presented model faithfully replicates primary colon tumorigenesis and follows classical signs of partial EMT. As such, it represents a valuable tool for preclinical studies aimed at evaluating therapeutic responses in CRC.

Decreased K8 expression in primary tumor of UALCAN patients with CRC than normal colon, supports the protective role of K8 in the colon. K8 expression was already reduced at stage 1 in patients without nodal metastasis, suggesting that K8 downregulation is an early event in CRC development. One of the control TAM-treated CDX2P-CreER^T2^; Apc^flox/+^ mice in our study, which retained K8 expression and developed a single colon tumor, exhibited lower K8 levels in the tumor compared with the normal colon tissue. Notably, human patients with CRC in our study confirmed the UALCAN findings with decreased K8 expression in the tumor and its nearby areas in comparison to the normal colon. Similar finding has been reported where a reduction in K8 expression was seen in CRC tissue relative to normal colon.^49^ Interestingly, multiple K8 isoforms were elevated in the normal colon of patients with colon polyps or tumors, suggesting that K8 expression may increase in normal mucosa as adenomas progress to carcinomas.^83^ These findings align with our model, where the colon shows a distribution of K8-negative crypts adjacent to K8-positive crypts, potentially reflecting the diseased and normal epithelium seen in human CRC. Most studies and databases typically compare paired CRC tumors with normal tissue from the same patient, but it is critical to assess K8 expression across a broader spectrum—from healthy colon mucosa to morphologically normal tissue and CRC lesions. This is particularly important because morphologically normal tissue near a polyp or tumor may be precancerous and not truly representative of healthy colon tissue. Our data also suggests a lateral signaling effect from K8-negative crypts to neighboring K8-positive crypts, as the K8-positive crypts in TAM-treated mice were significantly taller and had more Ki67+ cells compared with vehicle-treated controls.

In conclusion, we present an improved colon-specific epithelial cell tumorigenesis mouse model with the following key features: (1) a local deficiency of K8 and partner keratins in colon epithelial cells, resulting in a mild colitis-like phenotype with epithelial damage, increased local inflammation, and a pro-proliferative crypt environment; (2) disrupted cell adhesion and altered apical-basolateral polarity; (3) stem and progenitor cell overpopulation; (4) asymmetric cell division; (5) early spontaneous development of an increased number of tumors in the colon epithelium when K8 downregulation is combined with monoallelic Apc loss, with (6) partial EMT and (7) a patchy, localized loss of K8 in the colon epithelium, with no effect on K8 levels in other organs, that mirrors the local changes observed in human CRC; and (8) a model that recapitulates the reduction of K8 expression seen in human colorectal tumors. These features make this model a promising candidate for preclinical CRC research, including drug testing. Importantly, this study enhances our understanding of the essential role of K8 in colonocyte protection of the epithelial barrier and its contribution to tumor suppression in the colon. The results herein also suggest that K8 has different roles in different parts of the colonic epithelium. In the proximal part, K8 protects from crypt damage and inflammation and is likely essential for tissue repair and regenerative processes, whereas in the distal parts, K8 has a more tumor suppressive function maintaining a balanced progenitor cell compartment and symmetric cell division.

## Materials and Methods

### Human Patients With CRC

Samples consisted of formalin-fixed, paraffin-embedded (FFPE) colectomy specimens from patients with CRC (n = 11 patients and 33 samples from different parts of the colon). Samples were obtained from the archives of the Pathology Department in Pori, Satakunta Wellbeing Services County, Finland via Auria Biobank, Turku, Finland, under permissions 115/2025 and AB25-6711.

### Experimental Mouse Models

To generate a colon-specific K8 knockdown mouse model, K8^flox/flox^ mice (C57BL/6) from Prof Karen M. Ridge (Northwestern University)^11^ were bred with CDX2P-CreER^T2^; Apc^flox/+^ mice (C57BL/6) provided by Prof Yatrik M. Shah (University of Michigan).^9^ The offsprings of K8^flox/+^; CDX2P-CreER^T2^; Apc^flox/+^ were crossed with K8^flox/flox^. The intercrosses of K8^flox/flox^; CDX2P-CreER^T2^; Apc^flox/+^ and K8^flox/flox^; Apc^flox/+^ generated both K8^flox/flox^; CDX2P-CreER^T2^ and K8^flox/flox^; CDX2P-CreER^T2^; Apc^flox/+^. Mice were housed at the Central Animal Laboratory of University of Turku and treated according to animal licenses (ESAVI/16359/2019 and ESAVI/4498/2023) approved by the State Provincial Office of South Finland.

Mice were genotyped with PuReTaq Ready-To-Go PCR Beads (Cytvia) using primers for K8 flox: (5′-GCGTGGCTTTGGGATTTAGATTAG-3′ and 5′-CCTCCAGCCATGTTTCTTTATCTC-3′), for Cre: (5′-AGTGCGTTCGAACGCTAGAGCCTGT-3′ and 5′- GAACCTGATGGACATGTTCAGG-3′) and for Apc flox: (Apc-P3: 5′-GTTCTGTATCATGGAAAGATAGGTGGTC-3′), (Apc-P4: 5′-CACTCAAAACGCTTTTGAGGGTTGATTC-3′) and (Apc-P5: 5′-GAGTACGGGGTCTCTGTCTCAGTGAA-3′).

### Tamoxifen Administration, Body Weight, and Stool Monitoring

TAM solution was prepared by dissolving TAM (Sigma-Aldrich) in vehicle (Sigma-Aldrich), and 100 mg/kg TAM was used. The vehicle solution contained only corn oil. Adult 2 to 3 months old K8^flox/flox^; CDX2P-CreER^T2^ and K8^flox/flox^; CDX2P-CreER^T2^; Apc^flox/+^ mice received a daily TAM (+TAM) or vehicle (–TAM) dose intraperitoneally for 5 consecutive days. Mice were monitored for disease activity including body weight, stool consistency, and rectal bleeding. Stool consistency was scored as normal = 1, formed but soft = 2, slightly loose = 3, and liquid = 4. Rectal bleeding was calculated as no blood = 0, small blood amount in stool = 1, blood all over the stool = 2, visible blood at rectal area = 3, and fresh bleeding = 4, as previously described.^35^ K8^flox/flox^; CDX2P-CreER^T2^ and K8^flox/flox^; CDX2P-CreER^T2^; Apc^flox/+^ mice were sacrificed 28 days and 78 days after the first TAM administration, respectively. CDX2P-CreER^T2^; Apc^flox/+^ mice were used as an additional control for K8^flox/flox^; CDX2P-CreER^T2^; Apc^flox/+^ mice. All experiments had an equal representation of males and females. For the time course analysis, K8^flox/flox^; CDX2P-CreER^T2^ mice were sacrificed on day 1, 5, and 10 after the first TAM administration. K8^flox/flox^; CDX2P-CreER^T2^; Apc^flox/+^ mice were sacrificed on days 35, 56, and 67 after the first TAM administration.

### Organoid Isolation and Culture

K8^flox/flox^; CDX2P-CreER^T2^ mice were euthanized by CO_2_ inhalation and distal colon was cut into 2-mm segments and washed 15 times with ice cold phosphate-buffered saline (PBS) pH 7.4 (Medicago) and then incubated with 2 mM ethylenediaminetetraacetic acid (EDTA) pH 8.0 (Merck) in PBS for 20 minutes at room temperature, after which they were resuspended in PBS. Individual crypts were mechanically isolated by shaking (by hand 5–10 times, pooling the released crypts), and then centrifuged 290 × g for 5 minutes at +4 °C. The pellet of crypts was resuspended in PBS and mixed with mouse IntestiCult Organoid Growth Medium (STEMCELL Technologies) and Matrigel (Corning) to get 100 crypts per 50 μL and 1:1 ratio of PBS-medium: Matrigel. Fifty μL of crypt suspension was pipetted into prewarmed wells with coverslips in a 24-well plate. After the Matrigel polymerized, IntestiCult Organoid Growth Medium supplemented with 10 μM Y-27632 ROCK inhibitor (Adooq Bioscience) and 50 U/mL and 50 μg/mL penicillin-streptomycin (Sigma-Aldrich) was added. After 24 hours, the crypts were treated with 1 μM TAM in EtOH or vehicle (EtOH) in IntestiCult Organoid Growth Medium supplemented with penicillin-streptomycin for 3 days, whereafter crypts were grown in IntestiCult Organoid Growth Medium. On day 8 after isolation, crypts were collected for immunofluorescence and spindle symmetry analysis.

### Tissue Collection and Processing

Mice were euthanized by CO_2_ inhalation, and whole colon was removed and washed in ice cold PBS pH 7.4 and colon length was measured. For CRC mouse models, colon tumors were identified and quantified under a preparative microscope Leica M60 (Leica). Proximal colon (PC) and distal colon (DC) tissues were collected for histology, immunoblotting, and immunohistochemistry analysis. Isolated tissues were either snap frozen and stored in liquid N_2_, fixed immediately in 4 % paraformaldehyde (PFA) (ThermoFischer Scientific) pH 7.4, followed by paraffin embedding, or embedded in optimal cutting temperature (OCT) compound (Sakura Finetek) and kept at −80 °C. Similarly, ileum tissue (approximately 2 cm distal from the cecum) was also collected for protein analysis.

### Histological Examination

Paraffin embedding, processing (4-μm thick sections), and hematoxylin and eosin (H&E) staining of 4 % PFA fixed tissue samples was performed by Histocore facility at Institute of Biomedicine, University of Turku. H&E-stained samples were scanned using Pannoramic 1000 slide scanner (3DHISTECH). Colon crypt lengths were measured, and % crypt loss was analyzed using CaseViewer 2.4 (3DHISTECH). H&E-stained individual crypts per mouse were quantified to determine mean crypt length. For each mouse, the colon mucosal regions along mucosae muscularis with prominent erosion and lack of crypts were quantitated as % crypt loss of the entire perimeter of colon.^11^

### Sodium Dodecyl Sulfate-Polyacrylamide Gel Electrophoresis and Immunoblotting

Isolated total tissue samples were homogenized in ice cold homogenization buffer (0.187 M Tris-HCl pH 6.8, 3 % sodium dodecyl sulfate [SDS], 5 mM EDTA) supplemented with 1 x complete protease inhibitor cocktail (Roche) and 1 mM phenylmethylsulfonyl fluoride (PMSF) (Sigma-Aldrich) on ice. Afterwards, protein concentrations of each sample were quantified with Pierce bicinchoninic acid (BCA) protein assay kit (Thermo Fisher Scientific). Samples were prepared as 5 μg protein/10 μL using 3 × Laemmli sample buffer (30 % glycerol, 3 % SDS, 0.1875 M Tris-HCl pH 6.8, 0.015 % bromophenol blue, and 3 % β-mercaptoethanol). The samples were loaded on 10 % SDS-polyacrylamide gels along with precision plus protein dual color standards (BIO-RAD) to determine the molecular weight of the separated proteins. Proteins were then transferred to polyvinylidene fluoride (PVDF) (Cytvia) membranes and immunoblotted for proteins of interest. The primary antibodies used were: rabbit anti-K7 (181598) from Abcam, rat anti-K8 (Troma I) from Developmental Studies Hybridoma Bank, mouse anti-K18 (61028) from Progen, rat anti-K19 (MABT913) from EMD Millipore Corporation, rabbit anti-K20 (97511) from Abcam, sheep anti-IL22BP (AF2376) from R&D Systems, mouse anti-STAT3 (9139) from Cell Signaling Technology, rabbit anti-phospho-STAT3 (Tyr705) (9145) from Cell Signaling Technology, rabbit anti-Villin (PA5-17290) from Invitrogen, rabbit anti-Sox9 (82630) from Cell Signaling Technology, rabbit anti-Smad2/3 (3102) Cell Signaling Technology, rabbit anti-phosho-Smad2 (Ser465/467) (3108) Cell Signaling Technology, rabbit anti-Smad4 (38454) Cell Signaling Technology, mouse anti-Twist (50887) from Abcam, rabbit anti-β-actin (4967) from Cell Signaling Technology, mouse anti-β-tubulin (T8328) from Sigma-Aldrich, and rat anti-Hsc70 (SPA-815) from Enzo Life Science. The secondary antibodies used were: anti-rabbit Alexa-Fluor 800 (A32735) from Invitrogen, anti-rat Alexa-Fluor 680 (A21096) from Invitrogen, anti-mouse Alexa-Fluor 800 (A32730) from Invitrogen, anti-rabbit IgG-horseradish peroxidase (HRP) (W401B) from Promega, anti-rat IgG-HRP (7077) from Cell Signaling Technology, anti-mouse IgG-HRP (NA931V) from GE Healthcare, and anti-sheep IgG-HRP (12-342) from Sigma-Aldrich. The HRP signals were detected using western lightning plus-ECL (Perkin Elmer) and visualized with iBright CL1000 imaging system (Invitrogen). The fluorescent signals were visualized with iBright FL1000 imaging system (Invitrogen). The protein bands were quantified using ImageJ software (National Institutes of Health) as previously described^84^ and normalized to their respective loading controls.

### Immunohistochemistry

Frozen OCT-embedded samples were processed (6-μm thick sections) using a Leica CM 1950 Research Cryostat (Leica Microsystems). Tissue sections were fixed with either 1 % or 4 % PFA in PBS (pH 7.4), for 15 minutes, and organoids were fixed with 4 % PFA for 20 minutes and ice-cold methanol for 10 minutes. Afterwards, all the samples were immunostained as previously described.^85^ The primary antibodies used were: rabbit anti-K7 (181598) from Abcam, rat anti-K8 (Troma I) from Developmental Studies Hybridoma Bank, rabbit anti-K18 (SAB4501665) from Sigma-Aldrich, rat anti-K19 (MABT913) from EMD Millipore Corporation, rabbit anti-CDX2 (EPR2764Y) from Cell Marque, rabbit anti-MPO (RB-373-A0) from Thermo Fisher Scientific, rabbit anti-Ki67 (16667) from Abcam, rabbit anti-Notch-1 (sc-6014) from Santa Cruz, rabbit anti-beta-catenin (32572) from Abcam, and rabbit anti-Sox9 (82630) from Cell Signaling Technology, anti-phospho-histone 3 (Ser10) (9701) from Cell Signaling Technology, and mouse anti-β-tubulin (T8328) from Sigma-Aldrich. The secondary antibodies used were: anti-rabbit Alexa-Fluor 488/546 (A21206/A10040) from Invitrogen, anti-rat Alexa-Fluor 488/568/647 (A21208/A11077/A78947) from Invitrogen, and anti-mouse Alexa-Fluor 488 (A21202). The nuclei were stained with 4′,6 diamidino-2-phenylindole (DAPI; Invitrogen) before mounting the stained samples with ProLong Gold antifade reagent (Invitrogen).

Paraffin-embedded samples were processed and immunolabelled for rat anti-K8 (Troma I) from Developmental Studies Hybridoma Bank, rabbit anti-vimentin (5741) from Cell Signaling Technology, and rat anti-E-cadherin (14-3249-82) from Invitrogen with hematoxylin counterstain by Histocore facility at Institute of Biomedicine, University of Turku. The human CRC tissue samples were stained using the Ventana BenchMark Ultra Plus automated stainer (Roche Diagnostics) with rabbit anti-K8 clone EP17 (AC-0007) from Cell Marque. Protein detection was carried out with the Ventana UltraView Detection Kit (Roche Diagnostics), which includes a universal multimer secondary antibody, 3, 3′-diaminobenzidine (DAB) as the chromogen, and hematoxylin as the counterstain. Imaging was done using Marianas Spinning disk confocal microscope (Intelligent Imaging Innovations), Pannoramic 1000, Pannoramic MIDI slide scanners (3DHISTECH), UFS scanner (Philips), and Leica Stellaris 8 Falcon FILM microscope (Leica).

### Image Analysis

ImageJ (National Institutes of Health) and QuPath v0.2.3^86^ software were used for image analysis.

#### K8^flox/flox^; CDX2P-CreER^T2^ mice

For overall analysis between –TAM and +TAM mice, areas inside the mucosae muscularis (towards lumen) of colon were drawn. For analysis of differences in K8-positive (K8+) and K8-negative (K8–) areas in +TAM mice, K8+ or K8– crypts were selected based on K8 immunofluorescence staining. The number of MPO+ and PHH3+ cells were counted using the counting tool in QuPath and presented as number of MPO+ or PHH3+ cells per mm^2^ epithelial area. Ki67 and Sox9 cell positivity, Notch-1 and beta-catenin signal intensity were quantified in ImageJ as previously described.^87^^,^^88^ For Ki67 and Sox9 quantification, the Otsu threshold method was used, and watershed separation was applied for overlapping nuclei. Total number of nuclei (based on DAPI immunofluorescence) and Ki67+ nuclei were determined using the Analyze particles tool with the following parameters (size = 10–infinity, circularity = 0–1) and presented as percentage of Ki67+ cells per total number of cells. Sox9+ nuclei were manually detected and presented as percentage of nuclear Sox9+ cells per total number of cells. For quantifying the fluorescence intensity of Notch-1 and beta-catenin, mean gray value, area, and integrated intensity and their background signals were measured and shown as fold change of mean fluorescence intensity corrected against the background signal. Beta-catenin fluorescence intensity was quantified in lateral and apical membranes of nonadjacent cell membranes (indicated in Figure 5*B*).

#### K8^flox/flox^; CDX2P-CreER^T2^; Apc^flox^^/+^ mice

K8 and vimentin positivity (based on DAB staining) in dysplastic areas and colon adenocarcinoma were determined using QuPath positive cell detection tool as previously described.^89^ For vimentin cell positivity, multiple regions containing epithelial cells in the normal colon and colon adenocarcinoma of +TAM mice were manually drawn. Vimentin levels were quantified by positive cell detection tool. Cells were identified using hematoxylin stain and vimentin using mean intensity of cellular DAB stain (with threshold parameters of lowest at 0.01, middle at 0.4, and highest at 0.6) and presented as percentage of vimentin+ cells per total cells in the area. For E-cadherin quantification, several regions with approximately same area in the normal colon and colon adenocarcinoma of +TAM mice were drawn. Mean intensity of E-cadherin expression (based on DAB staining) per pixel in an area was measured using add intensity features tool and presented as E-cadherin mean intensity per area.

#### Human patients with CRC

Detection of epithelial cells and measurement of DAB intensity were performed using QuPath v0.5.1. For each patient, areas were manually annotated: 1 tumor region, adjacent normal epithelial cells, and normal epithelial cells from a region further away from the tumor. Each annotated area contained at least 1000 epithelial cells in the normal regions and at least 2000 cells in the tumor regions. The cellular expression of K8 was quantified based on the mean cellular intensity of DAB staining and presented as K8 mean cellular DAB intensity.

#### Mitotic spindle angle measurements in K8^flox/flox^;CDX2P-CreER^T2^ mouse organoids

To define colonocyte cell division symmetry, the orientation of the mitotic spindle of dividing cells was analyzed in organoids. Mitotic cells in metaphase or anaphase orientation were identified with DAPI and β-tubulin staining, and K8-positive and K8-negative cells were identified by K8 staining. For mitotic spindle angle measurements, the first line was drawn on the basal side of the organoid and the second one from one spindle pole to the other (see Figure 8). The mitotic spindle angle was calculated based on the relative angle of the 2 lines, where a low angle value indicates symmetric division and a higher value, asymmetric division.

### TCGA Analysis

Transcriptional expression of K8 was determined in multiple comparisons on the TCGA-COAD dataset using UALCAN.^90^^,^^91^ K8 expression analysis was conducted between sample types (normal and primary tumor), histological subtypes (normal, adenocarcinoma, and mucinous adenocarcinoma), individual cancer stages (normal, stage 1, stage 2, stage 3, and stage 4), nodal metastasis status (normal, N0, N1, and N2), patient’s age in years (normal, 21–40, 41–60, 61–80, and 81–100) and gender (normal, male, and female).

### Statistical Analysis and Data Preparation

Results were analyzed and graphs were generated using Prism 9 (GraphPad Software), and figures were prepared in Adobe Illustrator 2024 (Adobe, Inc). The statistical significance between 2 groups was determined after unpaired *t*-test. In a comparison between 2 groups analyzed at multiple time points, 2-way analysis of variance (ANOVA) with Bonferroni’s post hoc test was utilized. The significance is indicated as ∗*P* < .05; ∗∗*P* < .01; ∗∗∗*P* < .001; and ∗∗∗∗*P* < .0001.
